# The temporal effect of platelet-rich plasma on pain and physical function in the treatment of knee osteoarthritis: systematic review and meta-analysis of randomized controlled trials

**DOI:** 10.1186/s13018-017-0521-3

**Published:** 2017-01-23

**Authors:** Longxiang Shen, Ting Yuan, Shengbao Chen, Xuetao Xie, Changqing Zhang

**Affiliations:** 1 Department of Orthopaedic Surgery, Shanghai Sixth People’s Hospital affiliated to Shanghai Jiaotong University School of Medicine, 600 Yishan Road, Shanghai, 200233 China; 2 Section of Clinical Epidemiology, Institute of Orthopaedic Traumatology affiliated to Shanghai Jiaotong University, 600 Yishan Road, Shanghai, 200233 China

**Keywords:** Platelet-rich plasma, Hyaluronic acid, Knee, Osteoarthritis, Systematic review

## Abstract

**Background:**

Quite a few randomized controlled trials (RCTs) investigating the efficacy of platelet-rich plasma (PRP) for treatment of knee osteoarthritis (OA) have been recently published. Therefore, an updated systematic review was performed to evaluate the temporal effect of PRP on knee pain and physical function.

**Methods:**

Pubmed, Embase, Cochrane library, and Scopus were searched for human RCTs comparing the efficacy and/or safety of PRP infiltration with other intra-articular injections. A descriptive summary and quality assessment were performed for all the studies finally included for analysis. For studies reporting outcomes concerning Western Ontario and McMaster Universities Arthritis Index (WOMAC) or adverse events, a random-effects model was used for data synthesis.

**Results:**

Fourteen RCTs comprising 1423 participants were included. The control included saline placebo, HA, ozone, and corticosteroids. The follow-up ranged from 12 weeks to 12 months. Risk of bias assessment showed that 4 studies were considered as moderate risk of bias and 10 as high risk of bias. Compared with control, PRP injections significantly reduced WOMAC pain subscores at 3, 6, and 12 months follow-up (*p* = 0.02, 0.004, <0.001, respectively); PRP significantly improved WOMAC physical function subscores at 3, 6, and 12 months (*p* = 0.002, 0.01, <0.001, respectively); PRP also significantly improved total WOMAC scores at 3, 6 and 12 months (all *p* < 0.001); nonetheless, PRP did not significantly increased the risk of post-injection adverse events (RR, 1.40 [95% CI, 0.80 to 2.45], *I* ^*2*^ = 59%, *p* = 0.24).

**Conclusions:**

Intra-articular PRP injections probably are more efficacious in the treatment of knee OA in terms of pain relief and self-reported function improvement at 3, 6 and 12 months follow-up, compared with other injections, including saline placebo, HA, ozone, and corticosteroids.

**Review registration:**

PROSPERO CRD42016045410. Registered 8 August 2016.

**Electronic supplementary material:**

The online version of this article (doi:10.1186/s13018-017-0521-3) contains supplementary material, which is available to authorized users.

## Background

Osteoarthritis (OA) is a major cause of knee disability involving cartilage damage related to an inadequate healing response in the inflammatory milieu [1]. Current non-surgical treatment modalities include physiotherapy, analgesia, non-steroidal anti-inflammatory drugs, and intra-articular injections, such as hyaluronic acid (HA), corticosteroids, or Ozone, with the purpose of reducing symptoms and improving joint function [2–4].

In the past decade, there has been an increasing interest in the use of autologous growth factors, such as intra-articular injections of platelet-rich plasma (PRP) for treatment of knee OA [5]. PRP is a fraction of whole blood and prepared by the centrifugation of autologous blood, thereby yielding a higher concentration of platelets than baseline values. The regenerative effect and anti-inflammatory potential of PRP in the tissue healing process have led to extensive investigation of PRP as a potential treatment for a variety of musculoskeletal indications, including OA [6–8].

A number of randomized controlled trials (RCTs) were reported with favourable outcomes of PRP injections [9–17]; several reviews, including systematic reviews and meta-analysis, have been published with conclusion that PRP was found to be an effective and safe orthobiologic in the treatment of knee OA compared with other intra-articular injections [18–28]. However, these reviewers also concluded that more RCTs, in particular high-quality studies, were still needed. Considering that prior reviews either included non-RCTs or only synthesized a small number of RCTs (less than 9) for analysis [18–28] and that quite a few more RCTs recently have been published [29–35], we believe that it is necessary to perform an updated systematic review and meta-analysis, if appropriate, to evaluate whether the evidence-based support for PRP treatment will be strengthened or compromised. Furthermore, a large number of studies may allow us to fully investigate the temporal effect of PRP specifically on knee pain and physical function.

## Methods

This systematic review was registered online in PROSPERO (registration number: CRD42016045410) and was performed following the guidelines of the PRISMA statement. The protocol and the PRISMA checklist were provided as Additional files 1 and 2, respectively.

### Inclusion and exclusion criteria

All published RCTs evaluating the efficacy and/or safety of PRP (or preparations including autologous platelet concentrate, autologous conditioned plasma, and plasma rich in growth factors) in the treatment of knee OA in human were eligible for inclusion. Only studies that included patients aged 18 years or older with symptomatic knee OA and had a minimum follow-up of 12 weeks were included. All studies had to include at least 1 control group treated by intra-articular agents other than PRP. The studies that PRP was used in combination with operations were excluded. Published abstracts of RCTs without complete data for analysis were also excluded.

### Primary and secondary outcomes

For data synthesis across studies, the primary outcome was the Western Ontario and McMaster Universities Arthritis Index (WOMAC) [36]. Specifically, the WOMAC pain subscores, physical function subscores, and total scores at 3, 6, and 12 months after treatment were recorded. The secondary outcome was the number of patients reporting adverse events.

### Search strategy

Two investigators performed a systematic search of Pubmed, Embase, Cochrane library, and Scopus independently on July 15, 2016 and updated on November 15, 2016. The search strategy was as follows: (platelet[text word] OR plasma[text word]) AND (knee[text word] OR tibiofemoral[text word] OR patellofemoral[text word]) AND (*arthritis[text word] OR *arthritic[text word] OR cartilage[text word] OR *arthrosis[text word] OR gonarthrosis[text word]) AND random*[text word]. In Scopus, the search field [text word] was replaced with [TITLE-ABS-KEY]. No language or date exclusions were applied (Additional file 3).

Two investigators reviewed all titles and abstracts to remove duplicates and evaluate the relevance according to the inclusion and exclusion criteria. If ambiguity was encountered, the full-text review was performed. Any discrepancy was resolved through panel discussion with a third investigator. The references of prior systematic reviews were also reviewed to find potential eligible studies.

### Data extraction

Two reviewers independently performed data extraction using a pre-developed data extraction table. We extracted the basal characteristics of the included studies to form descriptive summaries. In multi-arm trials including more than one PRP treatment groups, only the group treated with at least twice PRP injections was considered as the intervention group, as the regimen of multiple PRP injections was more common and reported to be more efficacious than a single injection [37, 38]. Although data concerning the patients treated with single-PRP injection in those trials were also extracted, they were not used for quantitative synthesis. The extracted data were checked for consistency, and discrepancies were discussed until a consensus was reached. Personal correspondence was attempted to obtain missing data or clarify ambiguous information.

### Quality assessment

Two investigators independently assessed the methodological quality of each eligible study using Review Manager 5.3 (The Cochrane Collaboration, Oxford, England) to determine the risk of bias. The following domains were assessed: random sequence generation (selection bias), allocation concealment (selection bias), blinding of participants (performance bias), blinding of personnel (performance bias), blinding of outcome assessment (detection bias), incomplete outcome data (attrition bias), selective reporting (reporting bias), and other bias. The risk of bias for each domain was graded as either low (+), high (−), or unclear (?) [39]. A trial was regarded as low risk of bias only when all domains were scored as low risk of bias; if 1 or 2 domains were scored as high or moderate risk of bias, the trial was regarded as moderate risk of bias; if more than 2 domains were scored as high or moderate risk of bias, then high risk of bias was considered [21]. Differences were settled by panel discussion with a third investigator.

### Data analysis

For the continuous variables, the mean difference (MD) with 95% confidence interval (CI) was used, while the relative risk (RR) with 95% CI was adopted for dichotomous variables to express intervention effects. We assumed the presence of heterogeneity a priori and used the random-effects model in all pooled analysis. The *I* ^*2*^ was used to test heterogeneity. As defined previously, a value less than 40% means the heterogeneity might not be important, whereas the value more than 75% means considerable heterogeneity [39]. To detect the effect of individual studies on the pooled effect, sensitivity analysis was conducted. Publication bias was assessed with a funnel plot if there were at least 10 studies in a comparison [39]. Any *p* value less than 0.05 was considered to be statistically significant. All analysis was undertaken using Review Manager 5.3.

## Results

### Study characteristics

**Fig. 1:**
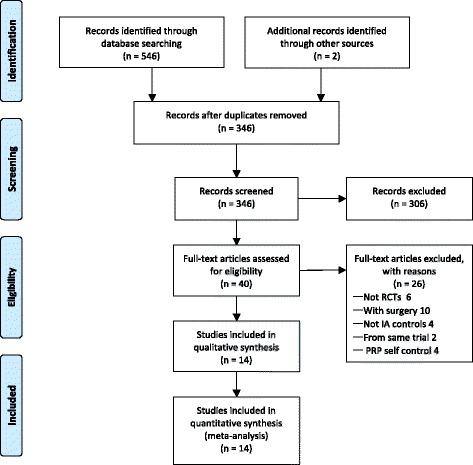
Study flow diagram

In total, 14 RCTs [9–11, 13–15, 17, 29–35] were included in the analysis published between 2011 and 2016. Details of the literature search were shown in a flowchart (Fig. 1). Search strategy and study selection process could be found in the Additional file 3.

A total of 1423 patients were included for randomization (Table 1). The sample size of PRP group ranged from 12 to 96 patients, whereas that of control groups including HA, placebo, ozone, and corticosteroids, ranged from 11 to 96 participants. WOMAC was the most commonly used efficacy outcome, and 9 studies reported WOMAC (8 studies) [9–11, 14, 15, 29, 34, 35] or normalized WOMAC (1 study) [13] scores. Follow-up intervals and length were variable among studies. The shortest follow-up was 12 weeks [32] and the longest was 12 months [15, 17, 29, 33, 34]. A summary of PRP intervention effect per study demonstrated comparable efficacy between PRP and HA among 215 patients in 2 studies [17, 32] and superior results in PRP-treated patients compared with control among 1208 patients in the rest 12 studies [9–11, 13–15, 29–31, 33–35].

**Table 1:** Basic characteristics of included studies

Studies	Country	Sample size	Age (years) Mean ± SD	% female	Body mass index (kg/m^2^)	Outcome measurement	Follow-up	Dropout	Risk of bias	Conclusion^a^
Cerza et al.[9]	Single centre Italy	PRP 60 HA 60	PRP 66.5 ± 11.3 HA 66.2 ± 10.6	PRP 58% HA 53%	NR	WOMAC total scores, adverse events	4, 12, 24 weeks	PRP 0 HA 0	High	+
Duymus et al.[29]	Single centre Turkey	PRP 41 HA 40 Ozone 39	PRP 60.4 ± 5.1 HA 60.3 ± 9.1 Ozone 59.4 ± 5.7	PRP 97% HA 97.1% Ozone 88.6%	PRP 27.6 ± 4.6 HA 28.4 ± 3.6 Ozone 27.6 ± 4.4	VAS, WOMAC scores	1, 3, 6, 12 months	PRP 8 HA 6 Ozone 4	High	+
Filardo et al.[17]	Single centre Italy	PRP 96 HA 96	PRP 53.3 ± 13.2 HA 57.6 ± 11.8	PRP 36.2% HA 41.6%	PRP 26.6 ± 4.0 HA 26.9 ± 4.4	IKDC subjective, KOOS, EQ-VAS, Tegner score, ROM, Transpatellar circumference, patient satisfaction, adverse events	2, 6, 12 months	PRP 2 HA 7	Moderate	–
Forogh et al.[30]	Single centre Iran	41 in total^b^	PRP 59.1 ± 7.0 CS 61.1 ± 6.7	PRP 70.8% CS 62.5%	PRP 28.9 ± 2.8 CS 29.2 ± 3.4	KOOS, VAS, ROM, 20 meters walk test, patient satisfaction	2, 6 months	PRP 1 CS 6	High	+
Görmeli et al.[31]	Single centre Turkey	PRP 46 PRP/S 45 HA 46 Placebo 45	PRP 53.7 ± 13.1 PRP/S 53.8 ± 13.4 HA 53.5 ± 14 Placebo 52.8 ± 12.8	PRP 58.9% PRP/S 56.8% HA 56.4% Placebo 50%	PRP 28.7 ± 4.8 PRP/S 28.4 ± 4.4 HA 29.7 ± 3.7 Placebo 29.5 ± 3.2	EQ-VAS, IKDC subjective, patient satisfaction	6 months	PRP 7 PRP/S 1 HA 7 Placebo 5	High	+
Li et al.[10]	Single centre China	PRP 15 HA 15	PRP 57.6 HA 58.2	PRP 60% HA 53.3%	PRP 24.3 HA 24	IKDC, WOMAC total score, Lequesne index, adverse events	3, 4, 6 months	PRP 0 HA 0	High	+
Montañez-Heredia et al.[35]	Single centre Spain	PRP 28 HA 27	PRP 66.3 ± 8.3 HA 61.5 ± 8.6	PRP 55.6% HA 65.4%	PRP 29.0 ± 5.5 HA 30.4 ± 4.9	VAS, KOOS, EUROQOL, adverse events	3, 6 months	PRP 1 HA 1	High	+
Patel et al.[11]	Single centre India	PRP_1_ 27 PRP_2_ 25 Placebo 26	PRP_1_ 53.1 ± 11.6 PRP_2_ 51.6 ± 9.2 Placebo 53.7 ± 8.2	PRP_1_ 59% PRP_2_ 80% Placebo 73.9%	PRP_1_ 25.8 ± 3.3 PRP_2_ 25.8 ± 3.3 Placebo 26.2 ± 2.9	WOMAC score, VAS, patient satisfaction, adverse events	6 weeks, 3, 6 months	PRP_1_ 1 PRP_2_ 0 Placebo 3	High	+
Paterson et al.[32]	Single centre Australia	PRP 12 HA 11	PRP 49.9 ± 13.7 HA 52.7 ± 10.3	PRP 27.3% HA 30%	PRP 27.9 ± 11.9 HA 30.9 ± 5.6	VAS, KOOS, KQoL, Functional tests, adverse events	4, 12 weeks	PRP 2 HA 2	Moderate	–
Raeissadat et al.[33]	Single centre Iran	PRP 87 HA 73	PRP 56.9 ± 9.1 HA 61.1 ± 7.5	PRP 89.6% HA 75.8%	PRP 28.2 ± 4.6 HA 27.0 ± 4.2	WOMAC total score, SF-36	52 weeks	PRP 10 HA 11	High	+
Sánchez et al.[13]	Multi-centre Spain	PRP 89 HA 87	PRP 60.5 ± 7.9 HA 58.9 ± 8.2	PRP 52% HA 52%	PRP 27.9 ± 2.9 HA 28.2 ± 2.7	Normalized WOMAC score, Lequesne index, adverse events	6 months	PRP 10 HA 13	Moderate	+
Smith et al.[34]	Single centre USA	PRP 15 Placebo 15	PRP 53.5 ± 8.2 Placebo 46.6 ± 9.4	PRP 66.7% Placebo 60%	PRP 29.5 ± 6.9 Placebo 27.5 ± 4.8	WOMAC score, adverse events	1, 2 weeks, 2, 3, 6, 12 months	PRP 0 Placebo 0	Moderate	+
Spaková et al.[14]	Single centre Slovakia	PRP 60 HA 60	PRP 52.8 ± 12.4 HA 53.2 ± 14.5	PRP 45% HA 48.3%	PRP 27.9 ± 4.1 HA 28.3 ± 4.0	WOMAC total score, NRS, adverse events	3, 6 months	PRP 0 HA 0	High	+
Vaquerizo et al.[15]	Multi-centre Spain	PRP 48 HA 48	PRP 62.4 ± 6.6 HA 64.8 ± 7.7	PRP 66.7% HA 54.2%	PRP 30.7 ± 3.6 HA 31.0 ± 4.6	WOMAC score, Lequesne index, adverse events	24, 48 weeks	PRP 0 HA 6	High	+

*NR* not reported, *VAS* visual analogue scale, *IKDC* international knee documentation committee, *KOOS* knee injury and osteoarthritis outcome score, *EQ-VAS* EuroQol VAS, *ROM* range of motion, *CS* corticosteroids, *PRP/S* single-PRP injection followed by saline injections, *EUROQOL* European quality of life scale, *PRP*_*1*_ single-PRP injections, *PRP*_*2*_ twice PRP injections, *KQoL* knee quality of life, *SF-36* short-form 36, *NRS* numeric rating scale

^a^+ comparison results favored PRP treatment; – comparison results did not favor PRP treatment

^b^The specific number of patients in each group was not described after randomization

PRP treatment protocols varied among studies in terms of preparation devices, centrifugations, the use of exogenous activators, and the injection regimen of dose, times, and intervals (Table 2).

**Table 2:** Details of PRP treatment protocols and control

	PRP						Control	
Studies	Category^a^	Preparation	Spinning	Activation	Injection dose, times, and intervals	Fresh/ frozen	Type	Injection dose, times, and intervals
Cerza et al.[9]	LP-PRP	ACP	Single	NR	5.5 mL, 4 times, weekly	Fresh	Hyalgan,	20 mg, 4 times, weekly
Duymus et al.[29]	LR-PRP	Ycellbio kit	Single	No	5 mL, 2 times, monthly	Fresh	Ostensil Plus, Ozone gas	40 mg, 1 time; 15 mL, 4 times, weekly
Filardo et al.[17]	LR-PRP	Custom	Double	CaCl_2_	5 mL, 3 times, weekly	Frozen	Hyalubrix,	30 mg, 3 times, weekly
Forogh et al.[30]	LR-PRP^b^	TUBEX kit	Double	CaCl_2_	5 mL, 1 time	Fresh	Depo Medrol	40 mg, 1 time
Görmeli et al.[31]^c^	LR-PRP	Custom	Double	CaCl_2_	5 mL, 3 times, weekly	1Fresh/ 2Frozen	Orthovisc, Saline	30 mg, 3 times, weekly; NR, 3 times, weekly
Li et al.[10]	LR-PRP	Weigao kit	Double	CaCl_2_	3.5 mL, 3 times, 3 weeks	Fresh	Sofast	2 mL, 3 times, 3 weeks
Montañez-Heredia et al.[35]	LP-PRP	Custom	Double	NR	NR, 3 times, 15 days	Frozen	Adant	NR, 3 times, 15 days
Patel et al.[11]^c^	LP-PRP	Custom	Single	CaCl_2_	8 mL, 2 times, 3 weeks	Fresh	Saline	8 mL, 1 time
Paterson et al.[32]	LR-PRP	Custom	Double	Ultraviolet	3 mL, 3 times, weekly	Fresh	Hylan G-F 20	3 mL, 3 times, weekly
Raeissadat et al.[33]	LR-PRP	Rooyagen kit	Double	No	4-6 mL, 2 times, 4 weeks	Fresh	Hyalgan	20 mg, 3 times, weekly
Sánchez et al.[13]	LP-PRP	PRGF-Endoret	Single	CaCl_2_	8 mL, 3 times, weekly	Fresh	Euflexxa	NR, 3 times, weekly
Smith et al.[34]	LP-PRP	ACP	Single	NR	3-8 mL, 3 times, weekly	Fresh	Saline	3-8 mL, 3 times, weekly
Spaková et al.[14]	LR-PRP	Custom	Triple	No	3 mL, 3 times, weekly	Fresh	Erectus	NR, 3 times, weekly
Vaquerizo et al.[15]	LP-PRP	PRGF-Endoret	Single	CaCl_2_	8 mL, 3 times, weekly	Fresh	Durolane	NR, 1 time

*ACP* autologous conditioned plasma, *NR* not reported, *CaCl*_*2*_ calcium chloride, *Depo Medrol* methylprednisolone acetate injectable suspension, *PRGF* plasma rich in growth factors

^a^ *PRP* was categorized into two types: LP-PRP (leukocyte-poor PRP) with the level of leukocytes below baseline and LR-PRP (leukocyte-rich PRP) with the level of leukocytes above baseline [45]

^b^Information was obtained from the authors through personal correspondence

^c^In a multi-arm trial, the group injected PRP more than once was regarded as an intervention group, and the data about the single-PRP injection group was not extracted

Among the 14 studies, 2 different radiographic OA grading systems were used: the Kellgren Lawrence grading (0–IV) [40] in 12 studies [9, 10, 14, 15, 17, 29–35] and the Ahlbäck scale (I–V) [41] in 2 studies [11, 13] (Table 3). According to the distribution of these cases, most participants receiving PRP treatment were at the early or mid-stage of knee OA.

**Table 3:** Radiographic OA grading

Studies	Intervention	Kellgren Lawrence					Ahlbäck		
		0	I	II	III	IV	I	II	III
Cerza et al.[9]	PRP		21	24	15				
	HA		25	22	13				
Duymus et al.[29]	PRP			22	11				
	HA			24	10				
	Ozone			23	12				
Filardo et al.[17]	PRP	0–IV, Mean ± SD: 2.0 ± 1.1							
	HA	0–IV, Mean ± SD: 2.0 ± 1.1							
Forogh et al.[30]^a^	PRP			7	17				
	CS			8	16				
Görmeli et al.[31]	PRP		I–III, 26			13			
	PRP/S HA		I–III, 30 I–III, 25			14 14			
	Placebo		I–III, 27			13			
Li et al.[10]	PRP		6	2	4	3			
	HA		6	3	3	3			
Montañez-Heredia et al.[35]	PRP		5	10	12				
	HA		2	9	15				
Patel et al.[11] ^a^	PRP_1_ PRP_2_						37 36	11 10	2 2
	Placebo						25	18	3
Paterson et al.[32]	PRP			II–III, 12					
	HA			II–III, 11					
Raeissadat et al.[33]	PRP		5	34	29	9			
	HA		0	29	23	10			
Sánchez et al.[13]	PRP						45	32	12
	HA						42	32	11
Smith et al.[34]	PRP			8	7				
	Placebo			10	5				
Spaková et al.[14]	PRP		2	39	19				
	HA		2	37	21				
Vaquerizo et al.[15]	PRP			14	26	8			
	HA			18	21	9			

*SD* standard deviation, *PRP/S* single-PRP injection followed by saline injections, *PRP1* single-PRP injection, *PRP2* twice PRP injections

^a^The number of knees rather than patients was reported

### Risk of bias assessment

**Fig. 2:**
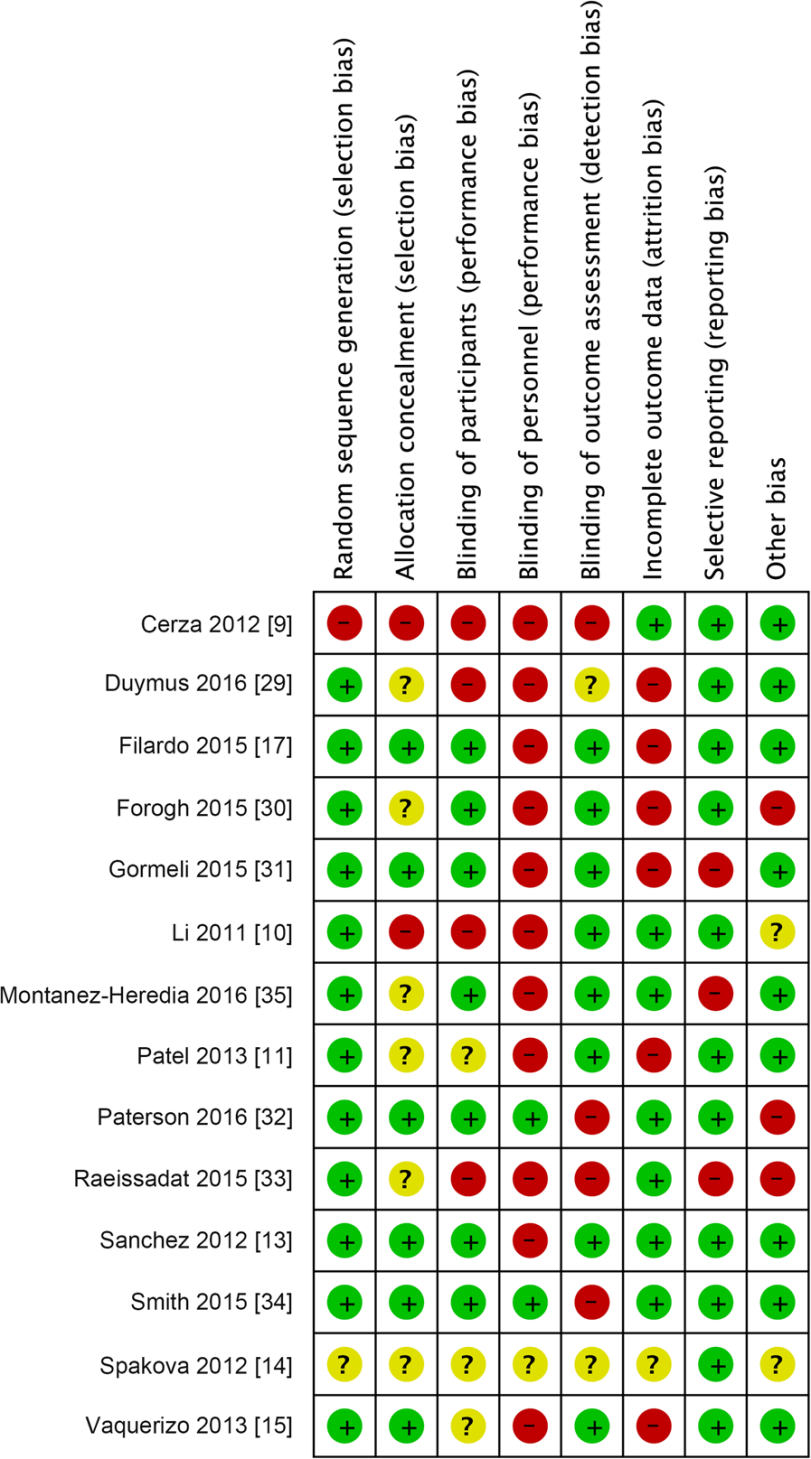
Risk of bias summary of all included studies. Methodological quality assessment of each study at 8 domains was illustrated. + means low risk of bias, ? means unclear risk of bias, and − means high risk of bias

A summary of risk of bias assessment of all included studies was illustrated in Fig. 2. Four studies [13, 17, 32, 34] achieved a moderate risk of bias, while the rest 10 [9–11, 14, 15, 29–31, 33, 35] obtained a high risk of bias (Table 1). A detailed justification of the evaluation of each domain of bias was described and provided in the Additional file 4.

### Knee pain

**Fig. 3:**
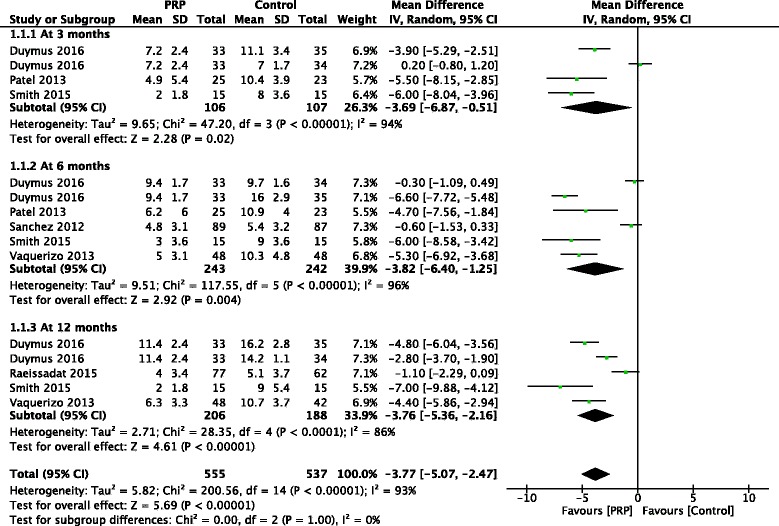
Forest plots investigating the effect of PRP on WOMAC pain subscores at 3, 6 and 12 months compared with control. (*IV*, inverse variance; *M-H*, Mantel-Haenszel; *CI*, confidence interval)

At 3 months, 3 studies reported WOMAC pain subscores, and a statistically significant difference was found in favor of PRP treatment compared with control (MD, −3.69 [95% CI, −6.87 to −0.51], *I* ^*2*^ = 94%, *p* = 0.02). At 6 months, the synthesis of 5 studies demonstrated a statistically significant difference in favor of PRP treatment (MD, −3.82 [95% CI, −6.40 to −1.25], *I* ^*2*^ = 96%, *p* = 0.004). At 12 months, the pooling results of 4 studies still favored PRP treatment (MD, −3.76 [95% CI, −5.36 to −2.16], *I* ^*2*^ = 86%, *p* < 0.001) (Fig. 3).

### Physical function

**Fig. 4:**
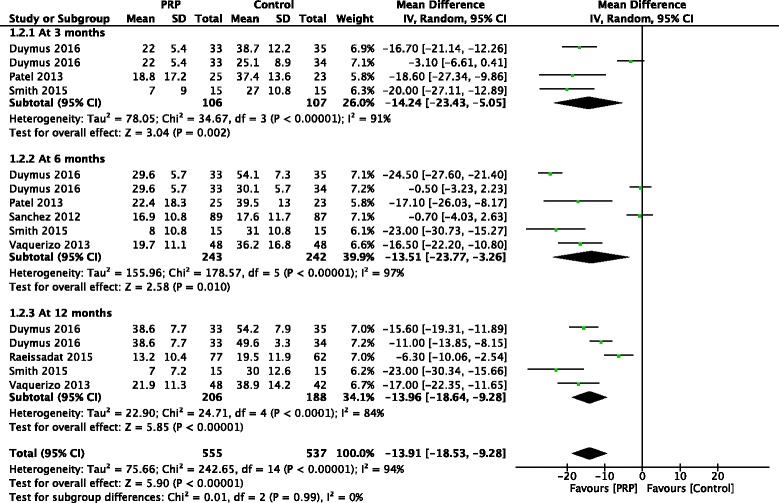
Forest plots investigating the effect of PRP on WOMAC physical function subscores at 3, 6, and 12 months compared with control. (*IV*, inverse variance; *M-H*, Mantel-Haenszel; *CI*, confidence interval)

At 3 months, 3 studies reported WOMAC physical function subscores, and a statistically significant difference was found in favor of PRP treatment compared with control (MD, −14.24 [95% CI, −23.43 to −5.05], *I* ^*2*^ = 91%, *p* = 0.002). PRP treatment was also found to improve physical function significantly according to the pooling analysis of 5 studies at 6 months (MD, −13.51 [95% CI, −23.77 to −3.26], *I* ^*2*^ = 97%, *p* = 0.01) and 4 studies at 12 months (MD, −13.96 [95% CI, −18.64 to −9.28], *I* ^*2*^ = 84%, *p* < 0.001) (Fig. 4).

### Total WOMAC scores

**Fig. 5:**
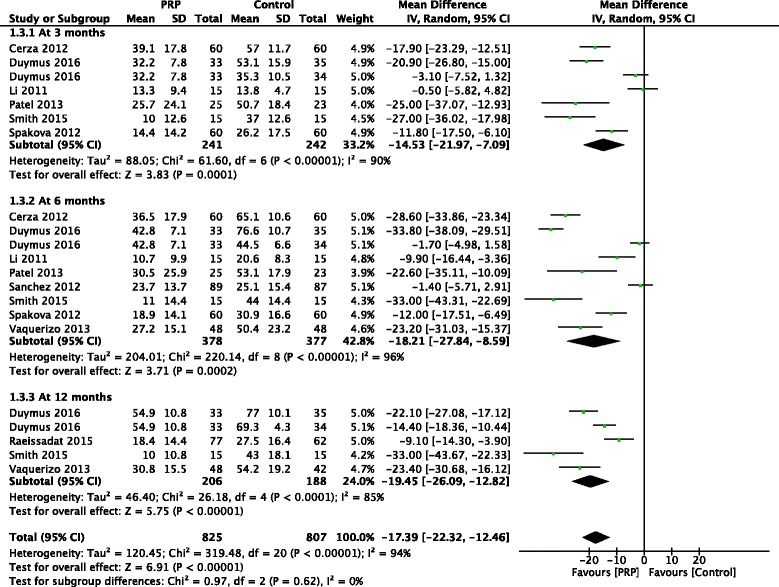
Forest plots investigating the effect of PRP on total WOMAC scores at 3, 6, and 12 months compared with control. (*IV*, inverse variance; *M-H*, Mantel-Haenszel; *CI*, confidence interval)

At 3 months, 6 studies reported total WOMAC scores and a statistically significant difference was found in favor of PRP treatment compared with control (MD, −14.53 [95% CI, −21.97 to −7.09], *I* ^*2*^ = 90%, *p* < 0.001). PRP treatment was also found to improve total WOMAC scores significantly according to the pooling analysis of 8 studies at 6 months (MD, −18.21 [95% CI, −27.84 to −8.59], *I* ^*2*^ = 97%, *p* < 0.001) and 4 studies at 12 months (MD, −19.45 [95% CI, −26.09 to −12.82], *I* ^*2*^ = 85%, *p* < 0.001) (Fig. 5).

### Adverse events

**Fig. 6:**
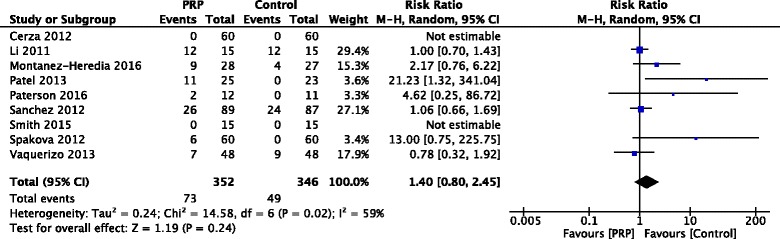
Forest plots comparing the risk of adverse events between PRP and control. (*M-H*, Mantel-Haenszel; *CI*, confidence interval)

A total of 10 studies [9–11, 13–15, 17, 32, 34, 35] recorded adverse events. Excluding the study by Filardo et al. [17], which reported adverse events in a different form, there was no statistically significant difference in the number of patients with adverse events between PRP and HA among the rest 9 studies (RR, 1.40 [95% CI, 0.80 to 2.45], *I* ^*2*^ = 59%, *p* = 0.24) (Fig. 6). All adverse events were non-specific, the symptoms including pain, stiffness, syncope, dizziness, headache, nausea, gastritis, sweating, and tachycardia. No severe complications were recorded and all the events were self-resolved in days.

## Discussion

This systematic review included 14 RCTs and assessed the temporal effect of PRP on knee pain and physical function in the treatment of knee OA compared with other intra-articular injections, including saline, HA, ozone, and corticosteroids. Data synthesis consistently showed intra-articular PRP injections significantly reduced knee pain, improved physical function, and total WOMAC scores compared with control. Such superiority was observed at 3, 6, and 12 months after treatment. However, the risk of adverse events in PRP-treated participants was not significantly increased in comparison with other intra-articular injections.

Although previous systematic reviews concluded that PRP was an effective and safe alternative to treat knee OA, such conclusion was reached on the basis of less than 9 RCTs [18–28], and thus the temporal effect of PRP injections on knee pain and physical function was not fully investigated. Chang et al. calculated the effect size of PRP treatment from different outcome measurements at 2, 6, and 12 follow-up, but half of the 16 studies included for analysis were case series, and 5 were RCTs [19]. Another systematic review pooled 6 RCTs and found that PRP obtained significantly better WOMAC total scores than HA from 3 to 12 months post-injection, however, only 2 studies reported WOMAC scores at 3 months and another 2 at 12 months [22]. Laudy et al. specifically evaluated the effect of PRP injections on knee pain and physical function at 6 and 12 months post-treatment [21]. Nonetheless, most comparisons included only 1 or 2 studies due to the small number of RCTs pooled for analysis. Another review included 9 RCTs and synthesized the WOMAC pain subscores and physical function subscores to compare the efficacy of PRP with control [23]. Due to the varied follow-up among studies, synthesis of the data at the latest follow-up might not reflect the changes of PRP efficacy. The strength of this study was to assess the effect of PRP treatment on knee pain and physical function at different time-points post-injection based on a larger number of RCTs.

It remains unclear regarding the duration period of the beneficial effect of PRP injections. Our study found that PRP was superior to other intra-articular injections in terms of pain relief and function improvement through 3 to 12 months. Filardo et al. investigated the persistence of the favorable effect of PRP infiltration during a 24-month follow-up [42]. Results show that all the evaluated parameters were significantly reduced at 24 months compared with those at 12 months, but still better than the baseline before treatment. The median duration of the clinical improvement was 9 months. This may explain why all current RCTs followed participants within 12 months. The short-term efficacy of PRP injections indicates that PRP only temporarily influences the joint milieu, without affecting the joint structure or progression of knee OA.

There are a few limitations in this review. The placebo effect was reportedly substantial in the treatment of knee OA, especially in terms of pain relief and self-reported function improvement [43]. Interventions that are recently “hot” or that were administered through needles, such as intra-articular injections, would result in larger placebo effect [44]. Therefore, blinding of participants is critical to minimize the potential placebo effect. Half of the 14 RCTs in this review were believed to have successfully performed blinding of participants [13, 17, 29–35] according to the risk of bias assessment. While 2 more studies [11, 15] stated blinding of participants, the difference in injection times between the intervention and control groups actually made it difficult to perform blinding reliably. So future RCTs should be designed as double-blinding, which ought to be performed successfully during the whole trials. Another limitation is the high heterogeneity among studies, which was also common in previous reviews [18–28].

## Conclusions

Intra-articular PRP injections probably are more efficacious in the treatment of knee OA in terms of pain relief and self-reported function improvement at 3, 6, and 12 months follow-up, compared with other injections, including saline placebo, HA, ozone, and corticosteroids.

## Additional files

Additional file 1: Intervention protocol. (PDF 119 kb)
Additional file 2: PRISMA checklist. (DOC 64 kb)
Additional file 3: Search strategy and study selection. (PDF 84 kb)
Additional file 4: Characteristics and risk of bias assessment of included and excluded studies. (PDF 462 kb)

## Abbreviations

CI: Confidence interval
CS: Corticosteroids
HA: Hyaluronic acid
IKDC: International knee documentation committee
KOOS: Knee injury and osteoarthritis outcome scores
LP-PRP: Leukocyte-poor PRP
LR-PRP: Leukocyte-rich PRP
MD: Mean difference
OA: Osteoarthritis
PRP: Platelet-rich plasma
RCTs: Randomized controlled trials
RR: Relative risk
WOMAC: Western Ontario and McMaster Universities Arthritis Index
VAS: Visual analogue scale
