# Supplementary material for: The temporal effect of platelet-rich plasma on pain and physical function in the treatment of knee osteoarthritis: systematic review and meta-analysis of randomized controlled trials
Source: J Orthop Surg Res. 2017 Jan 23;12:16. doi: 10.1186/s13018-017-0521-3 (PMC5260061; doi:10.1186/s13018-017-0521-3)
Supplement: Additional file 1: — Intervention protocol. (PDF 119 kb) [file 13018_2017_521_MOESM1_ESM.pdf]

UNIVERSITY of York
Centre for Reviews and Dissemination

NHS
National Institute for Health Research

PROSPERO International prospective register of systematic reviews

Platelet-rich plasma versus other intra-articular injections for treatment of knee osteoarthritis: a systemic review of randomized controlled trials

Xuetao Xie, Longxiang Shen, Ting Yuan, Shengbao Chen

Citation

Xuetao Xie, Longxiang Shen, Ting Yuan, Shengbao Chen. Platelet-rich plasma versus other intra-articular injections for treatment of knee osteoarthritis: a systemic review of randomized controlled trials. PROSPERO 2016:CRD42016045410 Available from
http://www.crd.york.ac.uk/PROSPERO_REBRANDING/display_record.asp?ID=CRD42016045410

Review question(s)

To assess the efficacy and safety of platelet-rich plasma versus other intra-articular injections for treatment of knee osteoarthritis

Searches

Database: PubMed, EMBASE, Cochrane Library and Scopus

potential search strategy: (platelet[text word] OR plasma[text word]) AND (knee[text word] OR tibiofemoral[text word] OR patellofemoral[text word]) AND (*arthritis[text word] OR *arthritic[text word] OR cartilage[text word] OR *arthrosis[text word] OR gonarthrosis[text word]) AND random*[text word]

Restrictions: limit to human; no language restrictions

Types of study to be included

Inclusions: randomized controlled studies

Exclusions: ongoing randomized controlled studies or those without complete data for analysis

Condition or domain being studied

knee osteoarthritis

Participants/ population

Inclusion criteria: patients aged 18 and over; with symptomatic knee degeneration

Exclusion criteria: history of surgical treatment of knee degeneration

Intervention(s), exposure(s)

Inclusion criteria: autologous platelet-rich plasma was injected into the osteoarthritic knee joints

Exclusion criteria: platelet-rich plasma was used in combination with surgery or other treatments considered as effective to relieve the symptoms of knee osteoarthritis

Comparator(s)/ control

Inclusion criteria: other intra-articular injections, such as placebo, hyaluronic acids, ozone, corticosteroids and so on.

Exclusion criteria: intra-articular injections in combination with other treatments considered as effective to relieve the symptoms of knee osteoarthritis

Outcome(s)

Primary outcomes

The knee scores at the last follow-up, measured by the Western Ontario and McMaster Universities Arthritis Index

Secondary outcomes
Adverse events;

Patient satisfaction

Risk of bias (quality) assessment
As suggested by the Cochrane Handbook for systemic reviews of interventions, two review authors will independently assess the risk of bias in included studies by considering the following characteristics:

random sequence generation (selection bias), allocation concealment (selection bias), blinding of participants (performance bias), blinding of personnel (performance bias), blinding of outcome assessment (detection bias), incomplete outcome data (attrition bias), selective reporting (reporting bias), and other bias.

Disagreements between the review authors over the risk of bias in particular studies will be resolved by discussion, with involvement of a third review author where necessary.

Strategy for data synthesis
We will present tables to provide a narrative synthesis of the findings from the included studies, structured around the type of intervention, target population characteristics, type of outcome and intervention content.

We anticipate that there will be limited scope for meta-analysis because of the range of different outcomes measured across the small number of existing trials. However, where studies have used the same type of intervention and comparator, with the same outcome measure, we will pool the results using a random-effects meta-analysis, with standardised mean differences for continuous outcomes and risk ratios for binary outcomes, and calculate 95% confidence intervals and two sided P values for each outcome. Heterogeneity between the studies in effect measures will be assessed using the I-squared statistic. We will consider an I-squared value greater than 50% indicative of substantial heterogeneity. We will conduct sensitivity analyses based on study quality. We will use stratified meta-analyses to explore heterogeneity in effect estimates according to: study quality; study populations; the logistics of intervention provision; and intervention content. We will also assess evidence of publication bias.

Analysis of subgroups or subsets
If the necessary data are available, subgroup analyses will be done for participants treated by different preparations of platelet-rich plasma, at different follow-ups, and with different controls. This is a systemic review including qualitative and quantitative synthesis and while subgroup analyses may be undertaken it is not possible to specify the groups in advance.

Contact details for further information
Dr Xie

600 Yishan Road, Shanghai, 200233 China

xuetaoxie@163.com

Organisational affiliation of the review
Shanghai Sixth People's Hospital

http://www.6thhosp.com/index.html

Review team
Dr Xuetao Xie, Shanghai Sixth People's Hospital
Dr Longxiang Shen, Shanghai Sixth People's Hospital
Dr Ting Yuan, Shanghai Sixth People's Hospital
Dr Shengbao Chen, Shanghai Jiaotong University

Anticipated or actual start date
01 July 2016

Anticipated completion date
15 September 2016

Funding sources/sponsors
National Natural Science Foundation of China (Grant No. 81401799); Shanghai Youth Science and Technology Start-up Grants (14YF1412100)

Conflicts of interest
None known

Language
English

Country
China

Subject index terms status
Subject indexing assigned by CRD

Subject index terms
Cartilage, Articular; Humans; Injections, Intra-Articular; Osteoarthritis, Knee; Platelet-Rich Plasma; Randomized Controlled Trials as Topic

Reference and/or URL for protocol
http://www.crd.york.ac.uk/PROSPEROFILES/45410_PROTOCOL_20160714.pdf

Stage of review
Completed but not published

Date of registration in PROSPERO
08 August 2016

Date of publication of this revision
15 August 2016

Stage of review at time of this submission	Started	Completed
Preliminary searches	Yes	Yes
Piloting of the study selection process	Yes	Yes
Formal screening of search results against eligibility criteria	Yes	Yes
Data extraction	Yes	Yes
Risk of bias (quality) assessment	Yes	Yes
Data analysis	Yes	Yes

PROSPERO
International prospective register of systematic reviews
The information in this record has been provided by the named contact for this review. CRD has accepted this information in good faith and registered the review in PROSPERO. CRD bears no responsibility or liability for the content of this registration record, any associated files or external websites.
