# Supplementary material for: The temporal effect of platelet-rich plasma on pain and physical function in the treatment of knee osteoarthritis: systematic review and meta-analysis of randomized controlled trials
Source: J Orthop Surg Res. 2017 Jan 23;12:16. doi: 10.1186/s13018-017-0521-3 (PMC5260061; doi:10.1186/s13018-017-0521-3)
Supplement: Additional file 3: — Search strategy and study selection. (PDF 84 kb) [file 13018_2017_521_MOESM3_ESM.pdf]

Additional file 3 Search Strategy and Study Selection

Search Date: Nov. 15, 2016

Pubmed

Search term: (platelet[text word] OR plasma[text word]) AND (Knee[text word] OR tibiofemoral[text word] OR patellofemoral[text word]) AND (*arthritis[text word] OR *arthritic[text word] OR cartilage[text word] OR *arthrosis[text word] OR gonarthrosis[text word]) AND random*[text word]

Limits to human

Search results: 40

Scopus

1960 to 2016

Search Term: (platelet[TITLE-ABS-KEY] OR plasma[TITLE-ABS-KEY]) AND (Knee[TITLE-ABS-KEY] OR tibiofemoral[TITLE-ABS-KEY] OR patellofemoral[TITLE-ABS-KEY]) AND (*arthritis[TITLE-ABS-KEY] OR *arthritic[TITLE-ABS-KEY] OR cartilage[TITLE-ABS-KEY] OR *arthrosis[TITLE-ABS-KEY]) AND random*[TITLE-ABS-KEY]

Search results: 224

Embase

1945 to 2016

Search term: (platelet[text word] OR plasma[text word]) AND (Knee[text word] OR tibiofemoral[text word] OR patellofemoral[text word]) AND (arthritis[text word] OR arthritic[text word] OR cartilage[text word] OR arthrosis[text word] OR osteoarthritis[text word] OR osteoarthritic[text word] OR gonarthrosis[text word]) AND random*[text word]

Without limits: 245

limits to human: 213

Search results: 213

Cochrane

Search term: (platelet[text word] OR plasma[text word]) AND (Knee[text word] OR tibiofemoral[text word] OR patellofemoral[text word]) AND (*arthritis[text word] OR *arthritic[text word] OR cartilage[text word] OR *arthrosis[text word] OR gonarthrosis[text word]) AND random*[text word]

Without limits: 191

limits to reviews: 69

Search results: 69

Total 40 + 224 + 213 + 69 = 546

Other sources: from reviews' references

2 （TITLE: Platelet-rich plasma intra-articular injections for cartilage degeneration and osteoarthritis: single- versus double-spinning approach.）(TITLE: Effect of single injection of platelet-rich plasma in comparison with corticosteroid on knee osteoarthritis: a double-blind randomized clinical trial)

Study Selection

Step 1: Remove Duplicates in EndNote X7: 202

After removal of duplicates 546 +2 – 202 = 346

Step 2: Read titles and abstracts, remove

due to Not PRP in OA 258

Not knee 3

Not human 4

Not article 41

After removal 346-258-3-4-41=40

Step 3: Read full-texts, remove

Not RCTs 6

with arthroplasty: 5

with arthroscopy 3

with osteotomy 2

Control group: oral treatment 2

physical therapy 2

PRP 4

Preliminary results 1

From another trial 1 (TITLE: Leukocyte-Rich Platelet-Rich Plasma Injections Do Not Up-Modulate Intra-Articular Pro-Inflammatory Cytokines in the Osteoarthritic Knee)

After removal 40-6-5-3-2-2-2-4-1-1=14

Final Analysis: 14 studies
