# Supplementary material for: The temporal effect of platelet-rich plasma on pain and physical function in the treatment of knee osteoarthritis: systematic review and meta-analysis of randomized controlled trials
Source: J Orthop Surg Res. 2017 Jan 23;12:16. doi: 10.1186/s13018-017-0521-3 (PMC5260061; doi:10.1186/s13018-017-0521-3)
Supplement: Additional file 4: — Characteristics and risk of bias assessment of included and excluded studies. (PDF 462 kb) [file 13018_2017_521_MOESM4_ESM.pdf]

Additional file 4 Characteristics and risk of bias assessment of studies

Characteristics of included studies

1. Cerza 2012

Study type<br>Country<br>Treatment<br>Conflicts of interest	2-arm RCT<br>Single centre, Italy<br>PRP versus Hyaluronic Acid<br>No
Participants	Mean age: PRP 66.5y, HA 66.2y<br>%Female: PRP 58%, HA 53%<br>Mean disease duration: NR<br>Number randomized: 120<br>Follow-up: 4, 12 and 24 weeks<br><u>Inclusion:</u><br>Age: NR<br>Duration clinical symptoms: NR<br>Symptomatic OA of knee, radiological<br>**Kellgren Lawrence** grade I-III<br><u>Baseline values:</u><br>Kellgren Lawrence grade n(%)<br>I: PRP 21(35%), HA 25(42%)<br>II: PRP 24(40%), HA 22(37%)<br>III: PRP 15(25%), HA 13(21%)<br>WOMAC score mean (SD)<br>Total: PRP 79.6(9.5), HA 75.4(10.7)
Intervention	Intervention (n=60):<br>4 PRP (ACP) (type *LP-PRP*) intra-articular injections<br>Dose: 5.5 mL<br>Interval: weekly<br>Comparison (n=60)<br>4 HA (Hyalgan) intra-articular injections<br>Dose: 20mg/2mL<br>Interval: weekly
Outcomes	Primary outcome:<br>WOMAC total score (0-96)<br>Adverse events
Results	WOMAC total score mean (SD)<br>4 weeks: PRP 49.6 (17.7), HA 55.2 (12.3), P<0.001<br>12 weeks: PRP 39.1 (17.8), HA 57 (11.7), P<0.001<br>24 weeks: PRP 36.5 (17.9), HA 65.1 (10.6), P<0.001<br>Adverse events:<br>No short time side effects observed

Risk of bias

Bias	Authors' judgement	Support for judgement
Random sequence generation (selection bias)	High risk	Quote: "patients were consecutively randomized… by their admission to our hospital"<br>Comment: Inappropriate
Allocation concealment (selection bias)	High risk	Quote: "patients were consecutively randomized… by their admission to our hospital"<br>Comment: Probably not done
Blinding of participants (performance bias)	High risk	Quote: "Groups allocated to the treatment with ACP underwent herochromocytometric examination"<br>Comment: Difficult to blind participants due to the difference in pre-treatment examination
Blinding of personnel (performance bias)	High risk	Quote: "The injections were performed by the unblinded physicians"<br>Comment: Probably not done
Blinding of outcome assessment (detection bias)	Unclear risk	Comment: Insufficient information
Incomplete outcome data (attrition bias)	Low risk	Number of allocated and analysed participants were reported. No patients withdrew or were excluded from analysis.
Selective reporting (reporting bias)	Low risk	Pre-specified outcomes were reported.
Other bias	Low risk	Power analysis were calculated.<br>Comment: unable to find other sources of bias

2. Duymus 2016

Study type<br>Country<br>Treatment<br>Conflicts of interest	3-arm RCT<br>Single centre, Turkey<br>PRP versus HA versus Ozone<br>NR

Participants	Mean age: PRP 60.4y, HA 60.3, Ozone 59.4<br>%female: PRP 97%, HA 97.1%, Ozone 88.6%<br>Mean disease duration: >1y<br>Number randomized: 120<br>Follow-up: 1, 3, 6 and 12 months<br><u>Inclusion:</u><br>Age: 47-80y<br>BMI: <30<br>Symptomatic OA of knee, radiological<br>**Kellgren Lawrence** grade II-III<br><u>Baseline values:</u><br>Kellgren Lawrence grade n(%)<br>II: PRP 22(66.7%), HA 24(61.8%), Ozone 23(65.8%)<br>III: PRP 11(33.3%), HA 10(38.2%), Ozone 12(34.2%)<br>VAS mean (SD)<br>PRP 7.4 (1.0), HA 8.3 (0.4), Ozone 7.2 (1.1)<br>WOMAC score mean (SD)<br>Pain: PRP 15.4(2.0), HA 16.6(1.1), Ozone 16.0(2.7)<br>Stiffness: PRP 6.1(0.9), HA 6.0(0.8), Ozone 6.4(1.0)<br>Physical function: PRP 54.5(6.7), HA 54.3(1.8), Ozone 53.5(8.7)<br>Total: PRP 76.1(9.4), HA 77.0(2.5), Ozone 76.0(11.9)
Intervention	Intervention (n=41):<br>2 PRP (type *LR-PRP*) intra-articular injections<br>Dose: 5 mL<br>Interval: monthly<br>Comparison 1 (n=40)<br>1 HA (40mg fermentative HA + 10mg mannitol) intra-articular injections<br>Dose: 40mg/2mL<br>Comparison 2 (n=39)<br>4 Ozone intra-articular injections<br>Dose: 15 mL<br>Concentration: 30 µg/mL<br>Interval: weekly
Outcomes	Primary outcome:<br>VAS (0-10)<br>WOMAC pain score (0-20), stiffness score (0-8), physical function score (0-68), total score (0-96)

Results	VAS mean (SD)<br>1 month: PRP 2.5(0.7), HA 2.6(1.2), Ozone 3.5(1.5), P<0.001<br>3 months: PRP 2.9(0.7), HA 3.1(0.9), Ozone 5.7(1.2), P<0.001<br>6 months: PRP 4.0(1.3), HA 4.3(1.3), Ozone 7.3(1.0), P<0.001<br>12 months: PRP 5.1(1.3), HA 6.8(0.1), Ozone 7.6(1.1), P<0.001<br>WOMAC pain score mean (SD)<br>1 month: PRP 6.8(1.8), HA 6.1(2.4), Ozone 6.6(3.5), P>0.05<br>3 months: PRP 7.2(2.4), HA 7 (1.7), Ozone 11.1(3.4), P<0.001<br>6 months: PRP 9.4(1.7), HA 9.7(1.6), Ozone 16.0(2.9), P<0.001<br>12 months: PRP 11.4(2.4), HA 14.2(1.1), Ozone 16.2(2.8), P<0.001<br>WOMAC stiffness score mean (SD)<br>1 month: PRP 2.8(0.8), HA 2.7(1.1), Ozone 2.7(1.6), P>0.05<br>3 months: PRP 3.0(1.1), HA 3.2(1.0), Ozone 4.2(1.3), P>0.05<br>6 months: PRP 3.6(0.7), HA 3.8(1.1), Ozone 6.4(1.0), P<0.001<br>12 months: PRP 4.7(1.2), HA 5.4(0.7), Ozone 6.5(0.1), P<0.001<br>WOMAC physical function score mean (SD)<br>1 month: PRP 19.7(7.1), HA 24.3(9.5), Ozone 21.7(8.6), P>0.05<br>3 months: PRP 22(5.4), HA 25.1(8.9), Ozone 38.7(12.2), P<0.001<br>6 months: PRP 29.6(5.7), HA 30.1(5.7), Ozone 54.1(7.3), P<0.001<br>12 months: PRP 38.6(7.7), HA 49.6(3.3), Ozone 54.2(7.9), P<0.001<br>WOMAC total score mean (SD)<br>1 month: PRP 26.4(9.5), HA 33.2(12.2), Ozone 31.1(12.9), P>0.05<br>3 months: PRP 32.2(7.8), HA 35.3(10.5), Ozone 53.1(15.9), P<0.001<br>6 months: PRP 42.8(7.1), HA 44.5(6.6), Ozone 76.6(10.7), P<0.001<br>12 months: PRP 54.9(10.8), HA 69.3(4.3), Ozone 77.0(10.1), P<0.001

Risk of bias

Bias	Authors' judgement	Support for judgement
Random sequence generation (selection bias)	Low risk	Quote: "patients were randomly assigned… by a computer-based protocol"<br>Comment: Probably done
Allocation concealment (selection bias)	Unclear risk	Comment: No information
Blinding of participants (performance bias)	High risk	Comment: Difficult to blind participants due to the difference in injection frequency and colours.

Blinding of personnel (performance bias)	High risk	Comment: Difficult to blind physicians due to the difference in injection frequency and colours.
Blinding of outcome assessment (detection bias)	Unclear risk	Comment: No information
Incomplete outcome data (attrition bias)	High risk	Number of allocated and analysed participants were reported. Eight patients were excluded from analysis in PRP, 6 from HA, while 4 from Ozone.
Selective reporting (reporting bias)	Low risk	Pre-specified outcomes were reported.
Other bias	Low risk	Power analysis were calculated.<br>Comment: unable to find other sources of bias

3. Filardo 2015

Study type<br>Country<br>Treatment<br>Conflicts of interest	2-arm RCT<br>Single centre, Italy<br>PRP versus Hyaluronic Acid<br>Yes
Participants	Mean age: PRP 53.3y, HA 57.6y<br>%Female: PRP 36%, HA 42%<br>Mean disease duration: > 4months<br>Number randomized: 192<br>Follow-up: 2, 6 and 12 months<br>Inclusion:<br>Age: <80y<br>Duration clinical symptoms: > 4months<br>Symptomatic OA of knee, radiological<br>**Kellgren Lawrence** grade 0, I-III<br>Baseline values:<br>BMI mean (SD): PRP 26.6(4.0), HA 26.9(4.4)<br>Kellgren Lawrence grade mean (SD): PRP 2.0 (1.1), HA2.0 (1.1)<br>IKDC score mean (SD): PRP 52.4(14.1), HA 47.9(13)<br>Tegner score mean (SD): PRP 2.9(1.3), HA 2.8(1.3)

Intervention	Intervention (n=96):<br>3 PRP (type *LR-PRP*) intra-articular injections<br>Dose: 5 mL<br>Interval: weekly<br>Comparison (n=96)<br>3 HA (Hyalubrix) intra-articular injections<br>Dose: 30mg/2mL<br>Interval: weekly
Outcomes	IKDC subjective score (0-100)<br>KOOS score (0-100)<br>EQ-VAS (0-100)<br>Tegner score (0-10)<br>Range of motion of knee<br>Transpatellar circumference<br>Patient satisfaction<br>Adverse events

Results	IKDC subjective score mean (SD)<br>2 months: PRP 63.2 (16.6), HA 63.5(15.2), P>0.05<br>6 months: PRP 65.0(16.1), HA 63.5(17.1), P>0.05<br>12 months: PRP 66.2(16.7), HA 64.2(18.0), P>0.05<br>KOOS symptom score mean (SD)<br>2 months: PRP 72.9 (17.0), HA 70.9(16.6), P>0.05<br>6 months: PRP 74.7(16.9), HA 72.7(17.4), P>0.05<br>12 months: PRP 73.9(17.2), HA 73.9(18.4), P>0.05<br>KOOS pain score mean (SD)<br>2 months: PRP 73.8 (19.9), HA 72.6(17.9), P>0.05<br>6 months: PRP 74.7(19.3), HA 74.8(17.6), P>0.05<br>12 months: PRP 74.9(19.3), HA 75.4(19.0), P>0.05<br>KOOS ADL score mean (SD)<br>2 months: PRP 79.0 (19.8), HA 78.0(17.9), P>0.05<br>6 months: PRP 79.1(19.6), HA 78.4(18.6), P>0.05<br>12 months: PRP 78.4(20.7), HA 78.4(19.3), P>0.05<br>KOOS sport score mean (SD)<br>2 months: PRP 48.0(26.1), HA 44.0(25.5), P>0.05<br>6 months: PRP 49.6(28.6), HA 45.1(27.0), P>0.05<br>12 months: PRP 49.3(28.6), HA 46.3(28.1), P>0.05<br>KOOS QOL score mean (SD)<br>2 months: PRP 48.4(23.1), HA 47.7(22.1), P>0.05<br>6 months: PRP 49.2(23.4), HA 49.9(23.1), P>0.05<br>12 months: PRP 50.8(24.0), HA 50.9(24.4), P>0.05<br>EQ-VAS score mean (SD)<br>2 months: PRP 76.3(12.7), HA 73.9(13.7), P>0.05<br>6 months: PRP 76.2(12.9), HA 74.1(15.1), P>0.05<br>12 months: PRP 77.6(11.1), HA 73.4(15.2), P>0.05<br>Tegner score mean (SD)<br>2 months: PRP 3.6(1.4), HA 3.3(1.5), P>0.05<br>6 months: PRP 3.7(1.5), HA 3.5(1.5), P>0.05<br>12 months: PRP 3.7(1.3), HA 3.4(1.5), P>0.05<br>Range of motion, degree mean (SD)<br>2 months: PRP 130.6(11.8), HA 129.0(10.9), P>0.05<br>6 months: PRP 130.3(10.7), HA 128.0(11.4), P>0.05<br>12 months: PRP 130.2(11.1), HA 127.4(12.0), P>0.05<br>Transpatellar circumference, mm mean (SD)<br>2 months: PRP 411.4(35.2), HA 413.3(34.1), P>0.05<br>6 months: PRP 407.2(35.6), HA 408.7(32.5), P>0.05<br>12 months: PRP 402.3(33.4), HA 406.4(33.6), P>0.05<br>Patient satisfaction rate: PRP 88.3%, HA 89.9%<br>Adverse events:<br>No severe adverse events<br>PRP injections produced significantly more post-injection swelling and pain with respect to HA

Risk of bias

Bias	Authors' judgement	Support for judgement
Random sequence generation (selection bias)	Low risk	Quote: "randomization list was provided by an independent statistician"<br>Comment: Probably done
Allocation concealment (selection bias)	Low risk	Quote: "sealed envelopes containing the treatment allocation"<br>Comment: Probably done
Blinding of participants (performance bias)	Low risk	Quote: "syringe was appropriately covered to prevent patients from discovering the substance"<br>Further: blood was harvested from each patient<br>Comment: Probably done
Blinding of personnel (performance bias)	High risk	Quote: "To guarantee the double-blinding of the trial, all evaluations were performed by an independent physician not involved in the injection procedure" indicating the physicians administering the injections were not blinded<br>Comment: Probably not done
Blinding of outcome assessment (detection bias)	Low risk	Quote: "To guarantee the double-blinding of the trial, all evaluations were performed by an independent physician not involved in the injection procedure"<br>Comment: Probably done
Incomplete outcome data (attrition bias)	High risk	Number of allocated and analysed participants were reported.<br>Two patients were excluded from analysis in PRP, while 7 from HA.
Selective reporting (reporting bias)	Low risk	Pre-specified outcomes were reported.
Other bias	Low risk	Power analysis were calculated.<br>Comment: unable to find other sources of bias

4. Forogh 2015

Study type<br>Country<br>Treatment<br>Conflicts of interest	2-arm RCT<br>Single centre, Iran<br>PRP versus Corticosteroid (CS)<br>NR

Participants	Mean age: PRP 59y, CS 61y<br>%Female: PRP 71%, HA 63%<br>Mean disease duration: NR<br>Number randomized: 41<br>Follow-up: 2 and 6 months<br>Inclusion:<br>Age: 50-70y<br>Duration clinical symptoms: NR<br>Symptomatic OA of knee, radiological<br>**Kellgren Lawrence** grade II-III<br>Baseline values:<br>BMI mean (SD): PRP 28.9(2.8), HA 29.2(3.4)<br>Kellgren Lawrence grade, n (%)<br>II: PRP 7 (29.2%), CS 8 (33.3%)<br>III: PRP 17 (70.8%), CS 16 (66.7%)<br>KOOS pain score, mean (SD)<br>PRP 45.8 (13.5), CS 52.3 (11.8)<br>KOOS symptom score, mean (SD)<br>PRP 55.2 (14.0), CS 54.6 (16.8)<br>KOOS ADL score, mean (SD)<br>PRP 51.9 (14.2), CS 46.1 (21.5)<br>KOOS Quality of life score, mean (SD)<br>PRP 7.4 (8.4), CS 5.1 (7.4)<br>KOOS sport score, mean (SD)<br>PRP 5.9 (6.8), CS 5.0 (7.1)<br>VAS, mean (SD): PRP 81.3 (13.4), CS 77.8 (13.8)<br>20 meters walk test, second, mean (SD)<br>PRP 16.33 (4.4), CS 19.34 (5.3)<br>Active flexion, degree, mean (SD)<br>PRP 98.6 (13.9), CS 95.6 (11.1)<br>Passive flexion, degree, mean (SD)<br>PRP 114.9 (13.3), CS 108.5 (9.8)<br>Flexion contracture, degree, mean (SD)<br>PRP 0.9 (2.4), CS 0.3 (1.2)
Intervention	Intervention (n=24 knees):<br>1 PRP (type LR-PRP)# intra-articular injections<br>Dose: 5 mL<br>Interval: not applicable<br>Comparison (n=24 knees)<br>1 CS (methylprednisolone acetate) intra-articular injections<br>Dose: 40mg/1mL<br>Interval: not applicable
Outcomes	KOOS score (0-100)<br>VAS (0-100)<br>Range of motion of knee<br>20 meters walk test<br>Patient satisfaction#

Results	KOOS symptom score mean (SD)<br>2 months: PRP 74.1 (18.6), CS 59.4 (14.7)<br>6 months: PRP 78.1 (8.0), CS 58.3 (16.4)<br>KOOS pain score mean (SD)<br>2 months: PRP 73.5 (15.0), CS 60.0(16.3)<br>6 months: PRP 78.0 (10.5), CS 54.4(20.4)<br>KOOS ADL score mean (SD)<br>2 months: PRP 75.4 (13.1), CS 55.1 (20.3)<br>6 months: PRP 74.9 (15.0), CS 62.9 (19.1)<br>KOOS sport score mean (SD)<br>2 months: PRP 13.3 (9.9), CS 10.6 (6.8)<br>6 months: PRP 11.3 (8.0), CS 11.6 (10.4)<br>KOOS QOL score mean (SD)<br>2 months: PRP 25.4 (19.2), CS 17.6 (12.6)<br>6 months: PRP 30.5 (15.3), CS 17.4 (11.0)<br>VAS score mean (SD)<br>2 months: PRP 45.1 (23.4), CS 65.3 (19.3)<br>6 months: PRP 44.6 (15.6), CS 72.5 (16.2)<br>20 meters walk test, second, mean (SD)<br>2 months: PRP 14.4 (3.3), CS 17.9 (4.9)<br>6 months: PRP 15.6 (3.4), CS 18.2 (5.5)<br>Active flexion, degree, mean (SD)<br>2 months: PRP 103.2 (12.2), CS 99.4 (11.3)<br>6 months: PRP 103.8 (12.5), CS 97.6 (10.9)<br>Passive flexion, degree, mean (SD)<br>2 months: PRP 115.8 (13.1), CS 119.8 (47.3)<br>6 months: PRP 114.6 (11.3), CS 106.1 (9.8)<br>Flexion contracture, degree, mean (SD)<br>2 months: PRP 0.9 (2.4), CS 0 (0)<br>6 months: PRP 0.9 (2.4), CS 0 (0)<br>Patient satisfaction #<br>PRP: 23 satisfied, 0 dissatisfied<br>CS: 14 satisfied, 2 dissatisfied

Risk of bias

Bias	Authors' judgement	Support for judgement
Random sequence generation (selection bias)	Low risk	Quote: "block randomization method"<br>Comment: Probably done
Allocation concealment (selection bias)	Unclear risk	Not reported<br>Comment: Insufficient information

Blinding of participants (performance bias)	Low risk	Quote: “all the syringes were prepared outside the room and covered to prevent patients from seeing the injectate”<br>Further: blood was harvested from each patient<br>Comment: Probably done
Blinding of personnel (performance bias)	High risk	Quote: “The present study was a double-blinded trial. Patients and the assessor were unaware of the treatment group” indicating the physicians administering the injections were not blinded<br>Comment: Probably not done
Blinding of outcome assessment (detection bias)	Low risk	Quote: “The present study was a double-blinded trial. Patients and the assessor were unaware of the treatment group”<br>Comment: Probably done
Incomplete outcome data (attrition bias)	High risk	Number of allocated and analysed participants were reported.<br>One patients were excluded from analysis in PRP, while 6 from CS.
Selective reporting (reporting bias)	Low risk	Pre-specified outcomes were reported.
Other bias	High risk	Power analysis were not calculated.<br>It seemed that the knees rather than the number of patients were randomized.

5. Görmeli 2015

Study type<br>Country<br>Treatment<br>Conflicts of interest	4-arm RCT<br>Single centre, Turkey<br>PRP versus PRP+saline versus HA versus Saline<br>No

Participants	Mean age: 53.5y (range 27-84y)<br>%female: 55.6%<br>BMI: 29.1 (range 19.8-38)<br>Mean disease duration: > 4 months<br>Number randomized: 182<br>Follow-up: 6 weeks, 3 and 6 months<br>Inclusion:<br>Age: NR<br>BMI: <30<br>Symptomatic OA of knee, radiological<br>**Kellgren Lawrence** grade I-IV<br>Baseline values:<br>Mean age: PRP 53.7y, PRP+saline 53.8y, HA 53.5y, Saline 52.8y<br>%female: PRP 58.9%, PRP+saline 56.8%,HA 56.4%,Saline 50%<br>BMI: PRP 28.7, PRP+saline 28.4, HA 29.7, Saline 29.5<br>Kellgren Lawrence grade n(%)<br>I-III: PRP 26(66.7%), PRP+saline 30(68.1%), HA 25(64.1%), Saline 27(67.5%)<br>IV: PRP 13(33.3%), PRP+saline 14(31.8%), HA 14(35.8%), Saline 13(32.5%)<br>EQ-VAS mean (SD)<br>PRP 50.3(5.2), PRP+saline 50.3(5.8), HA 50.5(4.6), Saline 50.2(4.5)<br>IKDC subjective score mean (SD)<br>PRP 40.4(5), PRP+saline 41.2(6.1), HA 40.6(4.5), Saline 40.4(4.3)
Intervention	Intervention (n=46):<br>3 PRP (type *LR-PRP*) intra-articular injections<br>Dose: 5 mL<br>Interval: monthly<br>Comparison 1 (n=45)<br>1 PRP + 2 saline intra-articular injections<br>Dose: NR<br>Interval: weekly<br>Comparison 2 (n=46)<br>3 HA (Orthovisc) intra-articular injections<br>Dose: 30mg/2mL<br>Interval: weekly<br>Comparison 3 (n=45)<br>3 Saline intra-articular injections<br>Dose: NR<br>Interval: weekly
Outcomes	EQ-VAS (0-100)<br>IKDC subjective score (0-100)<br>Adverse events<br>Patient satisfaction (satisfied, partially satisfied, not satisfied)

Results	EQ-VAS mean (SD)<br>6 months: PRP 71.4(10.8), PRP+saline 62.0(6.3), HA 60.8(7.2), Saline 48.0(5.1)<br>I-III: PRP 78.2(4.9), PRP+saline 64.7(5.0), HA 64.0(6.0), Saline 48.4(5.1)<br>IV: PRP 57.8(4.2), PRP+saline 56.4(5.1), HA 55.1(5.4), Saline 47.2(5.0)<br>IKDC subjective score mean (SD)<br>6 months: PRP 60.8(9.8), PRP+saline 50.2(6.7), HA 48.4(6.2), Saline 36.5(4.8)<br>I-III: PRP 66.9(4.9), PRP+saline 52.4(6.3), HA 50.7(5.6), Saline 36.6(5.4)<br>IV: PRP 48.6(3.7), PRP+saline 45.5(5.0), HA 44.4(5.3), Saline 36.3(3.5)<br>Adverse events: NR<br>Patient satisfaction rate<br>satisfied: PRP 76.9%, PRP+saline 72.7%, HA 64.1%, Saline 5%<br>partially satisfied: PRP 12.8%, PRP+saline 18.2%, HA 23.1%, Saline 15%<br>not satisfied: PRP 10.3%, PRP+saline 9.1%, HA 12.8%, Saline 80%

Risk of bias

Bias	Authors' judgement	Support for judgement
Random sequence generation (selection bias)	Low risk	Quote: "patients were randomly assigned into four groups by a computer-derived protocol"<br>Comment: Probably done
Allocation concealment (selection bias)	Low risk	Quote: "group assignments were only accessible to the study assistant and concealed from the patients and researchers"<br>Comment: Probably done
Blinding of participants (performance bias)	Low risk	Quote: "participants were blinded, subjected to a standardized IA injection protocol"<br>Further: blood was drawn from all patients<br>Comment: Probably done
Blinding of personnel (performance bias)	High risk	Quote: "each injection was performed by an unblinded physician"
Blinding of outcome assessment (detection bias)	Low risk	Quote: "The patients were evaluated … by the clinician who was blinded to the patients and the content of the injections"<br>Comment: Probably done

Incomplete outcome data (attrition bias)	High risk	Number of allocated and analysed participants were reported.<br>Seven patients were excluded from analysis in PRP, 1 from PRP+saline, 7 from HA, while 5 from Saline.
Selective reporting (reporting bias)	High risk	Not all pre-specified outcomes were reported.<br>Adverse events were not reported.
Other bias	Low risk	Power analysis were calculated.<br>Comment: unable to find other sources of bias

6. Li 2011

Study type<br>Country<br>Treatment<br>Conflicts of interest	2-arm RCT<br>Single centre, China<br>PRP versus Hyaluronic Acid<br>No
Participants	Mean age: PRP 57.6y, HA 58.2y<br>%Female: PRP 60%, HA 53%<br>Mean disease duration: > 4months<br>Number randomized: 30<br>Follow-up: 3, 4 and 6 months<br>Inclusion:<br>Age: NR<br>Duration clinical symptoms: > 4 months<br>Symptomatic OA of knee, radiological<br>**Kellgren Lawrence** grade I-IV<br>Baseline values:<br>Kellgren Lawrence grade n(%)<br>I: PRP 6(40%), HA 6(40%)<br>II: PRP 2(13%), HA 3(20%)<br>III: PRP 4(27%), HA 3(20%)<br>IV: PRP 3(20%), HA 3(20%)<br>IKDC subjective score mean (SD)<br>PRP 55.4(8.8), HA 57.5(9.4)<br>WOMAC score mean (SD)<br>Total: PRP 27.7(13.8), HA 30.9(13.9)<br>Lequesne Index mean (SD)<br>PRP 8.0(3.7), HA 9.3(2.9)

Intervention	Intervention (n=15):<br>3 PRP (type *LR-PRP*) intra-articular injections<br>Dose: 3.5 mL<br>Interval: triweekly<br>Comparison (n=15)<br>3 HA (Hyalgan) intra-articular injections<br>Dose: 2mL<br>Interval: triweekly
Outcomes	IKDC subjective score (0-100)<br>WOMAC total score (0-96)<br>Lequesne index （0-24）<br>Adverse events
Results	IKDC subjective score mean (SD)<br>3 months: PRP 71.3 (12.5), HA 70.1 (12.5), P=0.78<br>4 months: PRP 75.9 (13.5), HA 73.1 (9.9), P=0.52<br>6 months: PRP 76.4 (13.5), HA 63.2 (11.9), P<0.01<br>WOMAC total score mean (SD)<br>3 months: PRP 13.3 (9.4), HA 13.8 (4.7), P=0.85<br>4 months: PRP 12.9 (9.7), HA 12.5 (6.6), P=0.90<br>6 months: PRP 10.7 (9.9), HA 20.6 (8.3), P=0.01<br>Lequesne index mean (SD)<br>3 months: PRP 4.8 (2.4), HA 4.7 (2.0), P=0.87<br>4 months: PRP 3.3 (1.2), HA 3.7 (1.2), P=0.37<br>6 months: PRP 3.1 (1.0), HA 6.6 (2.1), P<0.01<br>Adverse events:<br>PRP 12 patients 31 injections, HA 12 patients 30 injections

Risk of bias*

Bias	Authors' judgement	Support for judgement
Random sequence generation (selection bias)	Low risk	Reply: "randomization was performed according to a random number chart"<br>Comment: Probably done
Allocation concealment (selection bias)	High risk	Reply: "Allocation concealment was not performed"
Blinding of participants (performance bias)	High risk	Reply: "No"
Blinding of personnel (performance bias)	High risk	Reply: "No"
Blinding of outcome assessment (detection bias)	Low risk	Reply: "Yes"

Incomplete outcome data (attrition bias)	Low risk	Number of allocated and analyzed participants were reported. No patients withdrew or were excluded from analysis.
Selective reporting (reporting bias)	Low risk	All pre-specified outcomes were reported.
Other bias	Unclear risk	Reply: “Power analysis were calculated”<br>Comment: the sample size was very small

7. Montañez-Heredia 2016

Study type<br>Country<br>Treatment<br>Conflicts of interest	2-arm RCT<br>Single centre, Spain<br>PRP versus Hyaluronic Acid<br>No
Participants	Mean age: PRP 66.3 ± 8.3y, HA 61.5 ± 8.6y<br>%Female: PRP 55.6%, HA 65.4%<br>Mean disease duration: NR<br>Number randomized: 55<br>Follow-up: 3 and 6 months<br><u>Inclusion:</u><br>Age: 40-80y<br>Duration clinical symptoms: NR<br>Symptomatic OA of knee, radiological<br>**Kellgren Lawrence** grade I-III<br><u>Baseline values:</u><br>Kellgren Lawrence grade n(%)<br>I: PRP 5(18.5%), HA 2(7.7%)<br>II: PRP 10(37%), HA 9(34.6%)<br>III: PRP 12(44.4%), HA 15(57.7%)
Intervention	Intervention (n=28):<br>3 PRP (type *LP-PRP*) intra-articular injections<br>Dose: NR<br>Interval: 15 days<br>Comparison (n=27)<br>3 HA (Adant) intra-articular injections<br>Dose: NR<br>Interval: 15 days
Outcomes	KOOS score (0-100)<br>VAS<br>EUROQOL(European Quality of Life)<br>Adverse events

Results	KOOS: No data provided.<br>VAS at 3 months compared to baseline<br>Number of 50% decrease: PRP 15, HA 8<br>VAS at 6 months compared to baseline<br>Number of 50% decrease: PRP 12, HA 11<br>EUROQOL at 3 months compared to baseline<br>Number of worsening: PRP 1, HA 3<br>Number of similar: PRP 13, HA 14<br>Number of improvement: PRP 13, HA 9<br>EUROQOL at 6 months compared to baseline<br>Number of worsening: PRP 2, HA 4<br>Number of similar: PRP 13, HA 13<br>Number of improvement: PRP 12, HA 9<br>Adverse events<br>number of patients: PRP 9, HA 4

Risk of bias

Bias	Authors' judgement	Support for judgement
Random sequence generation (selection bias)	Low risk	Quote: "Patients were assigned using a table of random numbers"<br>Comment: Probably done
Allocation concealment (selection bias)	Unclear risk	Not reported.<br>Comment: Insufficient information
Blinding of participants (performance bias)	Low risk	Quote: "The patient did not know what was being infiltrated, as the syringes for both groups were opaque."<br>Further: blood was drawn from all participants.<br>Comment: Probably done
Blinding of personnel (performance bias)	High risk	Not reported. Reporting "double blinded" means blinding of participants and observers<br>Comment: Probably not done
Blinding of outcome assessment (detection bias)	Low risk	Quote: "Researchers not involved in infiltration and blinded as to which group the patients were assigned carried out patient data collection."<br>Comment: Probably done
Incomplete outcome data (attrition bias)	Low risk	Number of allocated and analysed participants were reported. Reasons for missing data were reported. One participant was excluded for analysis in 1PRP group, and 1 from HA group.

Selective reporting (reporting bias)	High risk	All pre-specified outcomes were not reported.
Other bias	Low risk	Power analysis were calculated.<br>Comment: unable to find other sources of bias

8. Patel 2013

Study type<br>Country<br>Treatment<br>Conflicts of interest	3-arm RCT<br>Single centre, India<br>1PRP versus 2PRP versus Placebo<br>Yes
Participants	Mean age: 52.8, %female: 70.7%<br>Mean disease duration: NR<br>Number randomized: 78 (156 knees)<br>Follow-up: 6 weeks, 3 and 6 months<br><u>Inclusion:</u><br>Age: NR<br>Duration clinical symptoms: NR<br>Symptomatic OA of knee, radiological<br>**Albäck** grade 1-3<br><u>Baseline values:</u><br>Albäck grade, n of knees<br>1: 1PRP 37, 2PRP 36, Placebo 25<br>2: 1PRP 11, 2PRP 10, Placebo 18<br>3: 1PRP 2, 2PRP 2, Placebo 3<br>WOMAC score mean (SD)<br>Pain: 1PRP 10.17(3.82), 2PRP 10.62(3.73), Placebo 9.04(3.73)<br>Stiffness: 1PRP 3.06(2.08), 2PRP 3.50(2.09), Placebo 2.70(2.02)<br>Physical function: 1PRP 36.12(13.08), 2PRP 39.10(11.34), Placebo 38.80(12.44)<br>Total: 1PRP 49.56(17.83), 2PRP 53.20(16.18), Placebo 45.54(17.29)<br>VAS pain mean (SD)<br>1PRP 4.56(0.61), 2PRP 4.64(0.56), Placebo 4.57(0.62)

Intervention	Intervention 1 (n=27/54 knees):<br>1 PRP (type *LP-PRP*) intra-articular injections<br>Dose: 8 mL<br>Interval: not applicable<br>Intervention 2 (n=25/50 knees)<br>2 PRP (type *LP-PRP*) intra-articular injections injections<br>Dose: 8 mL<br>Interval: triweekly<br>Comparison (n=23/46 knees)<br>1 saline intra-articular injections<br>Dose: 8 mL<br>Interval: not applicable
Outcomes	Primary outcome:<br>WOMAC subscale pain (0-20)<br>Secondary outcome:<br>VAS (0-10)<br>WOMAC stiffness score (0-8), physical function score (0-68), total score (0-96)<br>Patient satisfaction (%) (satisfied, partly satisfied, not satisfied)<br>Adverse events

Results	WOMAC pain score mean (SD)#<br>6 weeks: 1PRP 4.26(4.6), 2PRP 4.38(4.2), Placebo 9.48(4.0)<br>3 months: 1PRP 3.74(5.0), 2PRP 4.88(5.4), Placebo 10.35(3.9)<br>6 months: 1PRP 5.0(5.8), 2PRP 6.18(6.0), Placebo 10.87(4.0)<br>WOMAC stiffness score mean (SD)#<br>6 weeks: 1PRP 2.12(1.7), 2PRP 2.28(2.1), Placebo 2.76(2.1)<br>3 months: 1PRP 1.76(1.8), 2PRP 2.00(2.0), Placebo 2.91(2.0)<br>6 months: 1PRP 2.10(2.0), 2PRP 1.88(2.1), Placebo 2.76(1.9)<br>WOMAC physical function score mean (SD)#<br>6 weeks: 1PRP 18.98(14.6), 2PRP 18.30(14.2), Placebo 34.54(13.5)<br>3 months: 1PRP 16.98(15.7), 2PRP 18.82(17.2), Placebo 37.43(13.6)<br>6 months: 1PRP 20.08(17.8), 2PRP 22.40(18.3), Placebo 39.46(13.0)<br>WOMAC total score mean (SD)#<br>6 weeks: 1PRP 25.36(20.5), 2PRP 24.96(19.9), Placebo 46.78(18.5)<br>3 months: 1PRP 22.48(22.1), 2PRP 25.70(24.1), Placebo 50.70(18.4)<br>6 months: 1PRP 27.18(24.9), 2PRP 30.48(25.9), Placebo 53.09(17.9)<br>VAS pain score mean (SD)<br>6 months: 1PRP 2.16(1.5), 2PRP 2.54(1.7), Placebo 4.61(0.7)<br>Patient satisfaction<br>satisfied rate %<br>6 months: 1PRP 67.3%, 2PRP 64%, Placebo 4.3%<br>partly satisfied rate %<br>6 months: 1PRP 7.7%, 2PRP 4%, Placebo 6.5%<br>not satisfied rate %<br>6 months: 1PRP 25%, 2PRP 25%, Placebo 89.1%<br>Adverse events<br>number of patients: 1PRP 6, 2PRP 11, Placebo 0

Risk of bias

Bias	Authors' judgement	Support for judgement
Random sequence generation (selection bias)	Low risk	Quote: “participants were randomly divided by computer-derived random charts”<br>Comment: Probably done
Allocation concealment (selection bias)	Unclear risk	Not reported.<br>Comment: Insufficient information

Blinding of participants (performance bias)	Unclear risk	Quote: “participants were blinded and subjected to a standardized injection protocol”<br>Further: blood was drawn from all participants.<br>However, the difference in injection times among groups make the blinding difficult.<br>Comment: inadequate information
Blinding of personnel (performance bias)	High risk	Not reported. Reporting “double blinded” means blinding of participants and observers<br>Comment: Probably not done
Blinding of outcome assessment (detection bias)	Low risk	Quote: “…by a blinded observer”<br>Comment: Probably done
Incomplete outcome data (attrition bias)	High risk	Number of allocated and analysed participants were reported. Reasons for missing data were reported. One participant was excluded for analysis in 1PRP group, while 3 from Placebo group.
Selective reporting (reporting bias)	Low risk	Pre-specified outcomes were reported.<br>Further information was obtained by email.
Other bias	Low risk	Power analysis were calculated.<br>Comment: unable to find other sources of bias

9. Paterson 2016

Study type<br>Country<br>Treatment<br>Conflicts of interest	2-arm RCT<br>Single centre, Austrialia<br>PRP versus Hyaluronic Acid<br>No

Participants	Mean age: PRP 49.9y, HA 52.7y<br>%Female: PRP 27%, HA 30%<br>Mean disease duration: NR<br>Number randomized: 23<br>Follow-up: 4 and 12 weeks<br>Inclusion:<br>Age: NR<br>Duration clinical symptoms: NR<br>Symptomatic OA of knee, radiological<br>**Kellgren Lawrence** grade II-III<br>Baseline values:<br>Kellgren Lawrence grade n(%): NR<br>VAS score mean (SD): PRP 48.09(23.75), HA 39.70(21.90)<br>KOOS score mean (SD)<br>Symptoms: PRP 48.70 (15.83), HA 62.14 (17.99)<br>Pain: PRP 57.07 (11.21), HA 70.00 (11.25)<br>Function: PRP 70.72 (13.64), HA 75.44 (12.42)<br>Sport: PRP 31.82 (20.40), HA 47.00 (28.69)<br>QoL: PRP 30.11 (18.92), HA 41.87 (13.51)<br>KQoL sore mean (SD)<br>Physical: PRP 57.72 (18.35), HA 71.16 (14.91)<br>Activity: PRP 59.09 (23.33), HA 75.50 (15.71)<br>Emotional: PRP 46.97 (26.69) , HA 58.75 (24.25)<br>Functional tests mean (SD)<br>Hops: PRP 46.64 (33.04), HA 55.50 (35.43)<br>Knee bends: PRP 19.45 (8.25), HA 20.50 (13.23)
Intervention	Intervention (n=11):<br>3 PRP (type *LR-PRP*) intra-articular injections<br>Dose: 3 mL<br>Interval: weekly<br>Comparison (n=10)<br>3 HA (Hylan) intra-articular injections<br>Dose: 3 mL<br>Interval: weekly
Outcomes	Primary outcome:<br>VAS (0-100)<br>KOOS (0-100/category)<br>KQoL<br>Functional tests (maximum hopping distance, number of knee bends in 30s)<br>Adverse events

Results	VAS score mean (SD)<br>4 weeks: PRP 19.64 (17.61), HA 12.90 (14.06)<br>12 weeks: PRP 36.89 (25.42), HA 14.13 (9.30)<br>KOOS symptom score mean (SD)<br>4 weeks: PRP 57.14 (20.33), HA 61.07 (26.86)<br>12 weeks: PRP 57.86 (22.76), HA 80.16 (8.40)<br>KOOS pain score mean (SD)<br>4 weeks: PRP 71.47 (16.67), HA 67.22 (25.55)<br>12 weeks: PRP 68.89 (15.76), HA 79.32 (9.33)<br>KOOS function score mean (SD)<br>4 weeks: PRP 79.27 (15.08), HA 79.12 (28.63)<br>12 weeks: PRP 78.68 (15.87), HA 90.03 (7.31)<br>KOOS sport score mean (SD)<br>4 weeks: PRP 40.46 (28.32), HA 46.50 (33.75)<br>12 weeks: PRP 41.00 (27.77), HA 64.44 (23.64)<br>KOOS QoL score mean (SD)<br>4 weeks: PRP 40.89 (27.55), HA 42.50 (21.21)<br>12 weeks: PRP 38.75 (28.38), HA 54.86 (9.77)<br>KQoL physical score mean (SD)<br>4 weeks: PRP 65.00 (18.14), HA 68.33 (27.54)<br>12 weeks: PRP 68.83 (18.64), HA 80.55 (13.46)<br>KQoL activity score mean (SD)<br>4 weeks: PRP 72.73 (16.79), HA 78.50 (29.16)<br>12 weeks: PRP 70.00 (22.23), HA 88.89 (7.41)<br>KQoL emotional score mean (SD)<br>4 weeks: PRP 58.71 (23.68), HA 67.08 (29.88)<br>12 weeks: PRP 58.75 (29.49), HA 75.00 (16.00)<br>Functional tests hopps mean (SD)<br>4 weeks: PRP 57.64 (41.36), HA 51.50 (39.49)<br>12 weeks: PRP 79.33 (34.17), HA 79.25 (38.04)<br>Functional tests knee bends mean (SD)<br>4 weeks: PRP 22.27 (8.37), HA 25.30 (16.60)<br>12 weeks: PRP 31.44 (7.96), HA 31.13 (15.63)<br>Adverse events:<br>2 participants had minor pain and swelling in PRP group.

Risk of bias

Bias	Authors' judgement	Support for judgement
Random sequence generation (selection bias)	Low risk	Quote: "participants were randomly allocated … using a computer generated randomization list"<br>Comment: Probably done

Allocation concealment (selection bias)	Low risk	Quote: “Group allocation and concealment was performed by an independent staff member not involved with the assessment of participants”<br>Comment: Probably done
Blinding of participants (performance bias)	Low risk	Quote: “This process ensured the blinding of both the patients and treating doctors”<br>Further: blood was drawn from all participants; identical looking syringe.<br>Comment: Probably done
Blinding of personnel (performance bias)	Low risk	Quote: “the syringe was then provided to the independent staff……either retained or discarded the syringe containing PA-PRP depending group allocation”<br>Quote: “This process ensured the blinding of both the patients and treating doctors”<br>Further: blood was drawn from all participants; identical looking syringe.<br>Comment: Probably done
Blinding of outcome assessment (detection bias)	High risk	Reporting “double-blind” means blinding of participants and physicians<br>Comment: Probably not done
Incomplete outcome data (attrition bias)	Low risk	Number of allocated and analysed participants were reported. Reasons for missing data were reported. Four patients excluded for analysis were evenly spread between groups.
Selective reporting (reporting bias)	Low risk	Pre-specified outcomes were reported.
Other bias	High risk	Power analysis were not calculated and the sample size was very small.

10. Raeissadat 2015

Study type<br>Country<br>Treatment<br>Conflicts of interest	2-arm RCT<br>Single centre, Iran<br>PRP versus Hyaluronic Acid<br>No

Participants	Mean age: PRP 56.8y, HA 61.1y<br>%Female: PRP 89.6%, HA 75.8%<br>Mean disease duration: >3 months<br>Number randomized: 160<br>Follow-up: 4, 24 and 52 weeks<br><u>Inclusion:</u><br>Age: 40-70y<br>Duration clinical symptoms: > 3 months<br>Symptomatic OA of knee, radiological<br>**Kellgren Lawrence** grade I-IV<br><u>Baseline values:</u><br>BMI: PRP 28.2, HA 27.0<br>Kellgren Lawrence grade %<br>I: PRP 6%, HA 0<br>II: PRP 44%, HA 47%<br>III: PRP 38%, HA 37%<br>IV: PRP 12%, HA 16%<br>WOMAC score mean (SD)<br>Pain: PRP 8.46(4.17), HA 6.19(3.82)<br>Stiffness: PRP 2.24(1.76), HA 1.88(1.72)<br>Physical function: PRP 28.91(12.63), HA 19.88(12.32)<br>Total: PRP 39.5(17.06), HA 28.69(16.69)<br>SF-36 mean (SD)<br>physical functioning: PRP 37.4(24.92), HA 43.66(22.3)<br>role limitations due to physical health: PRP 28.83(31.11), HA 28.62(36.17)<br>pain: PRP 49.9(24.77), HA 45.45(20.5)<br>general health: PRP 61.68(25.72), HA 61.37(19.14)<br>PCS-36: PRP 178.14(81.00), HA 180.4(68.52)<br>Emotional wellbeing: PRP 61.01(26.86), HA 57.74(21.24)<br>role limitations due to emotional problems: PRP 50.64(43.46), HA 51.61(46.13)<br>Vitality: PRP 54.25(24.95), HA 54.43(21.47)<br>social functioning: PRP 63.31(28.41), HA 60.64(27.86)<br>MCS-36: PRP 229.22(95.62), HA 226.43(97.39)
Intervention	Intervention (n=87):<br>2 PRP (type *LR-PRP*) intra-articular injections<br>Dose: 4-6 mL<br>Interval: 4 weeks<br>Comparison (n=73)<br>3 HA (Hyalgan) intra-articular injections<br>Dose: 20mg/2mL<br>Interval: weekly
Outcomes	Primary outcome:<br>WOMAC total score (0-120)<br>SF-36 (0-100/category)

Results	WOMAC pain score mean (SD)<br>52 weeks: PRP 4.03 (3.36), HA 5.08 (3.71), P<0.001<br>WOMAC stiffness score mean (SD)<br>52 weeks: PRP 1.19 (1.4), HA 2.14 (1.66), P<0.001<br>WOMAC physical function score mean (SD)<br>52 weeks: PRP 13.19 (10.39), HA 19.51 (11.9), P<0.001<br>WOMAC total score mean (SD)<br>52 weeks: PRP 18.44 (14.35), HA 27.46 (16.36), P<0.001<br>SF-36 physical functioning mean (SD)<br>52 weeks: PRP 56.82 (25.68), HA 44.29 (28.14), P<0.001<br>SF-36 role-physical mean (SD)<br>52 weeks: PRP 53.98 (38.84), HA 33.46 (41.96), P<0.001<br>SF-36 bodily pain mean (SD)<br>52 weeks: PRP 77.11 (19.56), HA 53.56 (27.89), P<0.001<br>SF-36 general health mean (SD)<br>52 weeks: PRP 68.60 (18.75), HA 60.73 (26.70), P<0.001<br>SF-36 PCS-36 mean (SD)<br>52 weeks: PRP 255.96 (77.59), HA 189.39 (103.73), P<0.001<br>SF-36 vitality mean (SD)<br>52 weeks: PRP 63.14 (26.66), HA 54.61 (26.07), P<0.001<br>SF-36 social functioning mean (SD)<br>52 weeks: PRP 79.38 (21.63), 63.3 (32.55), P<0.001<br>SF-36 role-emotional mean (SD)<br>52 weeks: PRP 45.19 (39.03), HA 45.19 (39.03), P=0.217<br>SF-36 mental health mean (SD)<br>52 weeks: PRP 70.25 (25.24), HA 56.45 (24.49), P<0.001<br>SF-36 MCS-36 mean (SD)<br>52 weeks: PRP 6269.92 (91.48) , HA 216.91 (100.9), P=0.002

Risk of bias

Bias	Authors' judgement	Support for judgement
Random sequence generation (selection bias)	Low risk	Quote: "by using random numbers table"<br>Comment: Probably done
Allocation concealment (selection bias)	Unclear risk	Not reported<br>Comment: Insufficient information
Blinding of participants (performance bias)	High risk	Quote: "Our study limitations include …… not being blind ……"<br>Comment: Probably not done
Blinding of personnel (performance bias)	High risk	Quote: "Our study limitations include …… not being blind ……"<br>Comment: Probably not done
Blinding of outcome assessment (detection bias)	High risk	Quote: "Our study limitations include …… not being blind ……"<br>Comment: Probably not done

Incomplete outcome data (attrition bias)	Low risk	Number of allocated and analyzed participants were reported. Reasons for missing data were reported and similar between groups. Ten patients excluded for analysis in PRP group while 11 from HA group.
Selective reporting (reporting bias)	High risk	Not all pre-specified outcomes were reported. Results at 4 and 24 weeks were not reported.
Other bias	High risk	Power analysis were not calculated. Significant difference existed between groups before treatment.

11. Sánchez 2012

Study type<br>Country<br>Treatment<br>Conflicts of interest	2-arm RCT<br>Multi-centre, Spain<br>PRP versus Hyaluronic Acid<br>No
Participants	Mean age: 59.7y, %Female: 51.7%<br>Mean disease duration: NR<br>Number randomized: 176<br>Follow-up: 1, 2 and 6 months<br>Inclusion:<br>Age: 40-72y<br>Duration clinical symptoms: NR<br>Symptomatic OA of knee, radiological<br>**Ahlbäck** grade I-III<br>Baseline values:<br>BMI: PRP 27.9, HA 28.2<br>Ahlbäck grade n(%)<br>I: PRP 45(51%), HA 42(49%)<br>II: PRP 32(36%), HA 32(38%)<br>III: PRP 12(13%), HA 11(13%)<br>WOMAC score, normalized mean (SD)<br>Pain: PRP 40.4(16), HA 38.4(5.6)<br>Stiffness: PRP 41.8(17.3), HA 38.5(18.3)<br>Physical function: PRP 39.6(16.3), HA 38.8(17.4)<br>Total: PRP 121.8(44.4), HA 115.6(45.1)<br>Lequesne index, mean (SD): PRP 9.5(3.0), HA 9.1(3.2)

Intervention	Intervention (n=89):<br>3 PRP (type *LP-PRP*) intra-articular injections<br>Dose: 8 mL<br>Interval: weekly<br>Comparison (n=87)<br>3 HA (Euflexxa) intra-articular injections<br>Dose: NR<br>Interval: weekly
Outcomes	<u>Primary outcome:</u><br>%of patients having a 50% decrease in the summed WOMAC pain subscale score<br><u>Secondary outcomes:</u><br>Normalized WOMAC total score (0-300)<br>Normalized WOMAC pain score (0-100)<br>Normalized WOMAC stiffness score (0-100)<br>Normalized WOMAC physical function score (0-100)<br>Lequesne index (0-24)<br>Adverse events
Results	50% decrease WOMAC pain score, n (%)<br>6 months: PRP 34(38.2%), HA 21(24.1%), P=0.044<br>Normalized WOMAC total score, mean (SD)<br>6 months: PRP 74.0(42.7), HA 78.3(48.1), P=0.561<br>Normalized WOMAC pain score, mean (SD)<br>6 months: PRP 24.1(15.5), HA 26.9(15.8), P=0.265<br>Normalized WOMAC stiffness score, mean (SD)<br>6 months: PRP 25.2(15.4), HA 25.5(17.9), P=0.901<br>Normalized WOMAC physical function score, mean (SD)<br>6 months: PRP 24.8(15.9), HA 25.9(17.2), P=0.682<br>Lequesne index, mean (SD)<br>6 months: PRP 5.2(3.4), HA 5.4(3.3), P=0.714<br>Adverse events, n: PRP: 26, HA 24, P=0.811

Risk of bias

Bias	Authors' judgement	Support for judgement
Random sequence generation (selection bias)	Low risk	Quote: "the treatment assigned by randomization was delivered. A stratified randomization (1 stratum per centre) was carried out"<br>Comment: Probably done
Allocation concealment (selection bias)	Low risk	Quote: "… keeping that relation in a sealed envelope"<br>Quote: "This envelope was not opened until the moment before applying the treatment"<br>Comment: Probably done

Blinding of participants (performance bias)	Low risk	Quote: “To maintain masking, the application area was hidden from view and blood was drawn from all patients …”<br>Comment: Probably done
Blinding of personnel (performance bias)	High risk	Quote: “Both the evaluators and patients remained blind to the treatments”<br>Reporting “double-blinded” means blinding of the evaluators and patients<br>Comment: Probably not done
Blinding of outcome assessment (detection bias)	Low risk	Quote: “Both the evaluators and patients remained blind to the treatments”<br>Quote: “Response was assessed by researchers not involved in the application of treatment. The data report forms did not make any references to the treatment applied”<br>Comment: Probably done
Incomplete outcome data (attrition bias)	Low risk	Number of allocated and analysed participants were reported. Reasons for missing data were reported and similar between groups.<br>Analysis: Intention to treat.
Selective reporting (reporting bias)	Low risk	All pre-specified outcomes were reported in the pre-specified way.
Other bias	Low risk	Power analysis were calculated.<br>Comment: unable to find other sources of bias.

12. Smith 2015

Study type<br>Country<br>Treatment<br>Conflicts of interest	2-arm RCT<br>Single centre, USA<br>PRP versus Placebo<br>Yes

Participants	Mean age: 50.06y, %Female: 63.3%<br>Mean disease duration: NR<br>Number randomized: 30<br>Follow-up: 1 and 2 weeks, 2, 3, 6 and 12 months<br>Inclusion:<br>Age: 30-80y<br>Duration clinical symptoms: >6 weeks<br>Symptomatic OA of knee, radiological<br>**Kellgren Lawrence** grade II-III<br>Baseline values:<br>BMI: PRP 29.53, Placebo 27.47<br>Kellgren Lawrence grade, n:<br>II: PRP 8, Placebo 10<br>III: PRP 7, Placebo 5<br>WOMAC score, mean (95%CI)<br>Pain: PRP 10 (9-11), Placebo 11 (10-12)<br>Stiffness: PRP 4 (4-5), Placebo 4 (4-5)<br>Physical function: PRP 32 (27-37), Placebo 31 (26-37)<br>Total: PRP 47 (41-53), Placebo 46 (40-53)
Intervention	Intervention (n=15):<br>3 PRP (type *LP-PRP*) intra-articular injections<br>Dose: 3-8 mL<br>Interval: weekly<br>Comparison (n=15)<br>3 Placebo (saline) intra-articular injections<br>Dose: 3-8 mL<br>Interval: weekly
Outcomes	Primary outcomes:<br>WOMAC subscales pain (0-20), stiffness (0-8), physical function (0-68) and total score (0-96)<br>Secondary outcomes:<br>Adverse events

Results	WOMAC pain score, mean (95%CI)<br>1 week: PRP 7 (6-8), Placebo 8 (6-10)<br>2 weeks: PRP 4 (2-6), Placebo 8 (6-9)<br>2 months: PRP 3 (1-5), Placebo 7 (5-9)<br>3 months: PRP 2 (1-4), Placebo 8 (6-9)<br>6 months: PRP 3 (1-4), Placebo 9 (7-11)<br>12 months: PRP 2 (1-4), Placebo 9 (6-11)<br>WOMAC stiffness score, mean (95%CI)<br>1 week: PRP 3 (2-4), Placebo 3 (2-4)<br>2 weeks: PRP 2 (1-3), Placebo 3 (2-4)<br>2 months: PRP 1 (1-2), Placebo 3 (2-3)<br>3 months: PRP 1 (0-2), Placebo 3 (2-4)<br>6 months: PRP 1 (0-2), Placebo 4 (3-5)<br>12 months: PRP 1 (0-2), Placebo 4 (3-5)<br>WOMAC physical function score, mean (95%CI)<br>1 week: PRP 24 (21-28), Placebo 27 (21-32)<br>2 weeks: PRP 16 (10-21), Placebo 25 (20-31)<br>2 months: PRP 9 (3-16), Placebo 22 (15-29)<br>3 months: PRP 7 (2-12), Placebo 27 (21-32)<br>6 months: PRP 8 (2-14), Placebo 31 (25-37)<br>12 months: PRP 7 (3-11), Placebo 30 (23-37)<br>WOMAC total score, mean (95%CI)<br>1 week: PRP 35 (29-40), Placebo 38 (30-46)<br>2 weeks: PRP 22 (15-29), Placebo 36 (28-44)<br>2 months: PRP 14 (5-23), Placebo 31 (22-41)<br>3 months: PRP 10 (3-18), Placebo 37 (30-45)<br>6 months: PRP 11 (3-20), Placebo 44 (36-53)<br>12 months: PRP 10 (4-17), Placebo 43 (33-54)<br>Adverse events, n: PRP 0, Placebo 0

Risk of bias

Bias	Authors' judgement	Support for judgement
Random sequence generation (selection bias)	Low risk	Quote: "used an automated, internet-based randomization system to ensure concealed randomization from the author and from eligible, consenting subjects"<br>The study was oversight by FDA.<br>Comment: Probably done
Allocation concealment (selection bias)	Low risk	Quote: "used an automated, internet-based randomization system to ensure concealed randomization from the author and from eligible, consenting subjects"<br>The study was oversight by FDA.<br>Comment: Probably done

Blinding of participants (performance bias)	Low risk	Quote: “This successfully established subject and investigator blinding”<br>This double-blind (patient and investigator) study was oversight by FDA.<br>Comment: Probably done
Blinding of personnel (performance bias)	Low risk	Quote: “This successfully established subject and investigator blinding”<br>This double-blind (patient and investigator) study was oversight by FDA.<br>Comment: Probably done
Blinding of outcome assessment (detection bias)	High risk	Not reported<br>This double-blind (patient and investigator) study was oversight by FDA.<br>Comment: Probably not done
Incomplete outcome data (attrition bias)	Low risk	Number of allocated and analysed participants were reported. No patient was excluded for analysis.
Selective reporting (reporting bias)	Low risk	All pre-specified outcomes were reported in the pre-specified way.
Other bias	Low risk	Despite the small sample size, the study was oversight by FDA and a post hoc power analysis proved adequate sample size, based on outcomes.

13. Spaková 2012

Study type<br>Country<br>Treatment<br>Conflicts of interest	2-arm RCT<br>Single centre, Slovakia<br>PRP versus Hyaluronic Acid<br>No

Participants	Mean age: 53y, %Female: 47.5%<br>Mean disease duration: NR<br>Number randomized: 120<br>Follow-up: 3 and 6 months<br><u>Inclusion:</u><br>Age: NR<br>Duration clinical symptoms: >6 months<br>Symptomatic OA of knee, radiological<br>**Kellgren Lawrence** grade I-III<br><u>Baseline values:</u><br>BMI: PRP 27.9, HA 28.3<br>Kellgren Lawrence grade, n:<br>I: PRP 2, HA 2<br>II: PRP 39, HA 37<br>III: PRP 19, HA 21<br>WOMAC total score, mean (SD)<br>Total: PRP 38.76(16.50), HA 43.21(13.70)<br>NRS, mean (SD): PRP 5.27(1.27), HA 6.02(1.77)
Intervention	Intervention (n=60):<br>3 PRP (type *LR-PRP*) intra-articular injections<br>Dose: 3 mL<br>Interval: weekly<br>Comparison (n=60)<br>3 HA (Erectus) intra-articular injections<br>Dose: NR<br>Interval: weekly
Outcomes	<u>Primary outcomes:</u><br>WOMAC (0-96)<br>NRS (0-11)<br><u>Secondary outcomes:</u><br>Adverse events
Results	WOMAC total score, mean (SD)<br>3 months: PRP 14.35 (14.18), HA 26.17 (17.47), P<0.01<br>6 months: PRP 18.85 (14.09), HA 30.90 (16.57), P<0.01<br>NRS, mean (SD)<br>3 months: PRP 2.06 (2.02), HA 3.98 (2.27), P<0.01<br>6 months: PRP 2.69 (1.86), HA 4.3 (2.07), P<0.01<br>Adverse events, n: PRP 6, HA 0

Risk of bias

Bias	Authors' judgement	Support for judgement

Random sequence generation (selection bias)	Unclear risk	Quote: “Patients were randomly divided into two groups”<br>No further information about randomization methods.<br>Comment: Insufficient information
Allocation concealment (selection bias)	Unclear risk	Not reported.<br>Comment: Insufficient information
Blinding of participants (performance bias)	Unclear risk	Not reported.<br>Comment: Insufficient information
Blinding of personnel (performance bias)	Unclear risk	Not reported.<br>Comment: Insufficient information
Blinding of outcome assessment (detection bias)	Unclear risk	Not reported.<br>Comment: Insufficient information
Incomplete outcome data (attrition bias)	Unclear risk	Not reported.<br>Comment: Insufficient information
Selective reporting (reporting bias)	Low risk	All pre-specified outcomes were reported.
Other bias	Unclear risk	Power analysis calculation was not reported.<br>Comment: Insufficient information

14. Vaquerizo 2013

Study type<br>Country<br>Treatment<br>Conflicts of interest	2-arm RCT<br>Multi-centre, Spain<br>PRP versus Hyaluronic Acid<br>Yes

Participants	Mean age: 63.6y, %Female: 60.4%<br>Mean disease duration: NR<br>Number randomized: 96<br>Follow-up: 24 and 48 weeks<br>Inclusion:<br>Age: >50y<br>Duration clinical symptoms: >6 months<br>Symptomatic OA of knee, radiological<br>**Kellgren Lawrence** grade II-IV<br>Baseline values:<br>Kellgren Lawrence grade n(%):<br>II: PRP 14(29.2%), HA 18(37.5%)<br>III: PRP 26(54.2%), HA 21(43.8%)<br>IV: PRP 8(16.7%), HA 9(18.8%)<br>WOMAC score, mean (SD)<br>Pain: PRP 9.6(2.5), HA 10.2(3.5)<br>Stiffness: PRP 3.7(1.7), HA 4.0(2.0)<br>Physical function: PRP 32.6(9.9), HA 36.7(13.7)<br>Total: PRP 45.9(12.7), HA 50.8(18.4)<br>Lequesne index, mean (SD): PRP 12.8(3.8), HA 13.1(3.8)
Intervention	Intervention (n=48):<br>3 PRP (type *LP-PRP*) intra-articular injections<br>Dose: 8 mL<br>Interval: biweekly<br>Comparison (n=48)<br>1 HA (Durolane) intra-articular injections<br>Dose: 60mg/3mL<br>Interval: Not applicable
Outcomes	Primary outcomes:<br>% of patients having a 30% decrease and 50% decrease in the summed WOMAC subscale scores—pain, stiffness and physical function and Lequesne index<br>Secondary outcomes:<br>WOMAC subscales pain (0-20), stiffness (0-8), physical function (0-68) and total score (0-96)<br>Lequesne scale (0-24)<br>Adverse events

Results	30% decrease WOMAC pain score, n(%)<br>24 weeks: PRP 40(83%), HA 7 (17%), P<0.001<br>48 weeks: PRP 28(58.3%), HA 5(11.9%), P<0.001<br>30% decrease WOMAC stiffness score, n(%)<br>24 weeks: PRP 24(52%), HA 11 (27%), P<0.02<br>48 weeks: PRP 24(52.2%), HA 5(12.2%), P<0.001<br>30% decrease WOMAC physical function score, n(%)<br>24 weeks: PRP 29(60%), HA 5 (11%), P<0.001<br>48 weeks: PRP 26(54.2%), HA 7(16.7%), P<0.001<br>50% decrease WOMAC pain score, n(%)<br>24 weeks: PRP 26(54%), HA 5 (11%), P<0.001<br>48 weeks: PRP 15(31%), HA 1(2%), P<0.001<br>50% decrease WOMAC stiffness score, n(%)<br>24 weeks: PRP 16(35%), HA 7 (16%), P=0.035<br>48 weeks: PRP 16(33%), HA 2(5%), P=0.001<br>50% decrease WOMAC physical function score, n(%)<br>24 weeks: PRP 19(40%), HA 5 (11%), P=0.001<br>48 weeks: PRP 15(31%), HA 0 (0), P=0.001<br>30% decrease Lequesne index, n(%)<br>24 weeks: PRP 35(73%), HA 7 (17%), P<0.001<br>48 weeks: PRP 23(47.9%), HA 1(2.4%), P<0.001<br>50% decrease Lequesne index, n(%)<br>24 weeks: PRP 14(29%), HA 9 (19%), P=0.002<br>48 weeks: PRP 2(4%), HA 1(2%), P=0.017<br>WOMAC pain score, mean (SD)<br>24 weeks: PRP 5.0 (3.1), HA 10.3 (4.8), P<0.001<br>48 weeks: PRP 6.3 (3.3), HA 10.7 (3.7), P<0.001<br>WOMAC stiffness score, mean (SD)<br>24 weeks: PRP 2.5 (1.7), HA 4.0 (2.3), P<0.001<br>48 weeks: PRP 2.6 (1.4), HA 4.7 (2.0), P<0.001<br>WOMAC physical function score, mean (SD)<br>24 weeks: PRP 19.7 (11.1), HA 36.2 (16.8), P<0.001<br>48 weeks: PRP 21.9 (11.3), HA 38.9 (14.2), P<0.001<br>WOMAC total score, mean (SD)<br>24 weeks: PRP 27.2 (15.1), HA 50.4 (23.2), P<0.001<br>48 weeks: PRP 30.8 (15.5), HA 54.2 (19.2), P<0.001<br>Lequesne index, mean (SD)<br>24 weeks: PRP 5.2 (3.4), HA 5.4 (3.3),<br>48 weeks: PRP 8.9 (3.7), HA 14.4 (3.8),<br>Adverse events, n: PRP 7, HA 9, P=0.610

Risk of bias

Bias	Authors' judgement	Support for judgement

Random sequence generation (selection bias)	Low risk	Quote: “The correspondence between the number of patients and their treatment was performed by use of specific software for randomization”<br>Comment: Probably done
Allocation concealment (selection bias)	Low risk	Quote: “… keeping that relation in a sealed envelope”<br>Quote: “This envelope was not opened until the moment before applying the treatment”<br>Comment: Probably done
Blinding of participants (performance bias)	Unclear risk	Quote: “To maintain masking, the application area was hidden from view and blood was drawn from all patients …”<br>However, the difference in injection times between groups make the blinding difficult.<br>Comment: inadequate information
Blinding of personnel (performance bias)	High risk	Quote: “Both the evaluators and patients remained blind to the assignment of treatments”<br>Reporting “double-blinded” means blinding of the evaluators and patients<br>Comment: Probably not done
Blinding of outcome assessment (detection bias)	Low risk	Quote: “Both the evaluators and patients remained blind to the assignment of treatments”<br>Quote: “response was assessed by researchers not involved in the application of treatment (blinded)”<br>Comment: Probably done
Incomplete outcome data (attrition bias)	High risk	Number of allocated and analysed participants were reported. Reasons for missing data were reported and similar between groups. But 6 patients were excluded for analysis in the HA group at 48 weeks.
Selective reporting (reporting bias)	Low risk	All pre-specified outcomes were reported in the pre-specified way.
Other bias	Low risk	Power analysis were calculated.<br>Comment: unable to find other sources of bias.

Characteristics of excluded studies

Study	Reasons for exclusion
Acosta-Olivo 2014	Control group: oral paracetamol
Angoorani 2015	Control group: Transcutaneous Electrical Nerve Stimulation
Filardo 2012 in BMC	A preliminary report of "Filardo 2015" study
Filardo 2012	Control group: another PRP preparation
Mariani 2016	Subjects from a published trial by "Filardo 2015"
Gobbi 2015	Control group: PRP only (with different frequency of injections)
Kavadar 2015	Control group: PRP only (with different frequency of injections)
Rayegani 2014	Control group: Therapeutic exercise

NR, not reported; ACP, autologous conditioned plasma; LP-PRP, leukocyte-poor PRP; BMI, body mass index; LR-PRP, leukocyte-rich PRP; NRS, numeric rating scale.

* Details concerning the study design were obtained from the authors.

# Data were obtained from the authors by email.
